# Differential alterations in gene expression profiles contribute to time-dependent effects of nandrolone to prevent denervation atrophy

**DOI:** 10.1186/1471-2164-11-596

**Published:** 2010-10-22

**Authors:** Weiping Qin, Jiangping Pan, William A Bauman, Christopher P Cardozo

**Affiliations:** 1 Center of Excellence for the Medical Consequences of Spinal Cord Injury1, Room 1E-02, James J. Peters VA Medical Center, 130 West Kingsbridge Road, Bronx, New York 10468, USA; 2 Department of Medicine, Mount Sinai School of Medicine, 1 Gustave L. Levy Place, New York, New York 10029, USA; 3 Department of Rehabilitation Medicine, Mount Sinai School of Medicine, 1 Gustave L. Levy Place, New York, New York 10029, USA

## Abstract

**Background:**

Anabolic steroids, such as nandrolone, slow muscle atrophy, but the mechanisms responsible for this effect are largely unknown. Their effects on muscle size and gene expression depend upon time, and the cause of muscle atrophy. Administration of nandrolone for 7 days beginning either concomitantly with sciatic nerve transection (7 days) or 29 days later (35 days) attenuated denervation atrophy at 35 but not 7 days. We reasoned that this model could be used to identify genes that are regulated by nandrolone and slow denervation atrophy, as well as genes that might explain the time-dependence of nandrolone effects on such atrophy. Affymetrix microarrays were used to profile gene expression changes due to nandrolone at 7 and 35 days and to identify major gene expression changes in denervated muscle between 7 and 35 days.

**Results:**

Nandrolone selectively altered expression of 124 genes at 7 days and 122 genes at 35 days, with only 20 genes being regulated at both time points. Marked differences in biological function of genes regulated by nandrolone at 7 and 35 days were observed. At 35, but not 7 days, nandrolone reduced mRNA and protein levels for FOXO1, the mTOR inhibitor REDD2, and the calcineurin inhibitor RCAN2 and increased those for ApoD. At 35 days, correlations between mRNA levels and the size of denervated muscle were negative for RCAN2, and positive for ApoD. Nandrolone also regulated genes for Wnt signaling molecules. Comparison of gene expression at 7 and 35 days after denervation revealed marked alterations in the expression of 9 transcriptional coregulators, including Ankrd1 and 2, and many transcription factors and kinases.

**Conclusions:**

Genes regulated in denervated muscle after 7 days administration of nandrolone are almost entirely different at 7 versus 35 days. Alterations in levels of FOXO1, and of genes involved in signaling through calcineurin, mTOR and Wnt may be linked to the favorable action of nandrolone on denervated muscle. Marked changes in the expression of genes regulating transcription and intracellular signaling may contribute to the time-dependent effects of nandrolone on gene expression.

## Background

Androgens have been shown to reverse muscle loss due to age [1,2], and to preserve muscle in persons with HIV infection [3,4] and burns [5,6]. In animal models, androgens also prevent or reduce atrophy due to disuse from spinal cord injury [7], immobilization [6], or unweighting [8]. The molecular basis for these beneficial effects remains poorly understood.

One major factor that contributes to muscle atrophy is accelerated catabolism of muscle proteins, which is largely attributable to the ubiquitin-proteasome pathway [9] and which has been linked to the muscle ubiquitin E3 ligases muscle atrophy F-box (MAFbx) and muscle Ring-finger 1 (MuRF1) [10-12]. Upregulation of MAFbx and MuRF1 has been attributed to activation of FOXO1 [13,14]. Degradation by MAFbx of the muscle differentiation factor MyoD [15] or the translation initiation factor eIF3F [16] have also been linked to muscle atrophy. In cardiac myocytes, MAFbx also reduces calcium-dependent signaling through calcineurin and has been shown to reduce myocyte size [17].

A role has been established in muscle atrophy for inhibitors of protein synthesis acting both up- and downstream of mTOR, a protein kinase that integrates signals regulating protein synthesis and cell size and has also been implicated in muscle hypertrophy [18,19]. Reductions in mTOR activity caused by dexamethasone or ethanol have been shown to be due to upregulation of REDD1 (also known as DDIT4 or RTP801) [20,21]. mTOR is also inhibited by REDD2 (also called DDIT4L or RTP801L), a protein closely related to REDD1 [22,23]. Expression of REDD1 is upregulated by FOXO1 [24], as is that of 4EBP1 [25], which inhibits translation by reducing the initiation of CAP-dependent translation by eIF4E [26].

We have recently found that administration for 7 days of the anabolic steroid nandrolone reduced denervation atrophy when begun 29 days after nerve transection (35 days) associated with reduced levels of mRNA for MAFbx and MuRF1, but without changes in expression of IGF-1, its receptor, or IGF-1 binding proteins 2, 3, 4 or 5 [27]. However, when begun at the time of denervation, administration of nandrolone for the same 7 day period (7 days) did not prevent atrophy or reduce expression of MAFbx or MuRF1 [27]. The molecular mechanisms by which nandrolone slows atrophy at 35 days are unclear. While MAFbx and MuRF1 accelerate denervation atrophy in mice [10] and degrade several proteins that determine muscle mass [15-17], their levels do not necessarily correlate to the response to interventions that spare muscle [28]. Thus, there are likely to be additional actions of nandrolone that contribute to its protective effects on denervated muscle at 35 days. In addition, the molecular determinants that prevent the anabolic actions of nandrolone at 7 days are unknown.

We reasoned that comparison of genes regulated by nandrolone at 7 and 35 days would permit identification of those genes regulated only at 35 days, and which are thus likely to be associated with protection against atrophy. Additionally, we predicted that the changes in gene expression in denervated muscle between 7 and 35 days formed the basis for the increased responsiveness to nandrolone at 35 days; because many actions of nandrolone involve its binding to the androgen receptor (AR), a transcription factor that is activated when drug or hormone are bound, we predicted that there were changes over this period in the expression of genes encoding factors that either promoted or prevented transcriptional activity of the AR at target genes. Here, we have tested these possibilities using oligonucleotide microarrays with verification of the expression of selected genes by real time PCR (qPCR) and Western blotting.

## Results

### Filtering of microarray data

**Figure 1:**
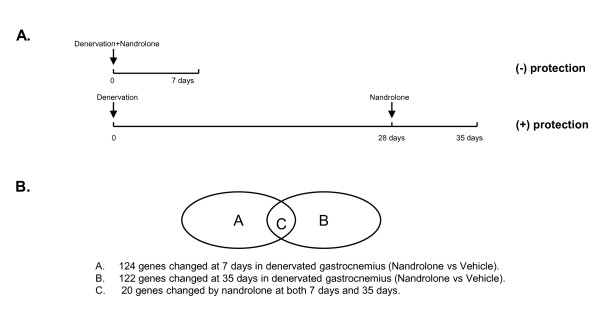
**Summary of the experimental design and of the overlap of genes regulated by nandrolone at 7 and 35 days**. **A**. Scheme of the experimental design. **B**. Venn diagram depicting the overlap of genes regulated by nandrolone at 7 and 35 days.

Probesets representing 124 known genes (**Pool A**) were altered by nandrolone in denervated gastrocnemius at 7 days after nerve transection (Figure 1B and Additional file 1). At 35 days, nandrolone changed the expression of 276 genes (**Pool D**, data not shown) in denervated gastrocnemius muscle.

Before comparing **Pools A and D**, we examined the possible confounding effects of changes in gene expression over time due to the effects of denervation on skeletal muscle. A comparison of gene expression in denervated gastrocnemius muscle from animals administered vehicle revealed 318 unique genes that were altered at day 35 as compared to day 7 (**Pool E**, Additional file 2). Among these, 154 were also present in **Pool D **and were altered in the same direction by time (35 versus 7 days) and nandrolone. These genes were removed from **Pool D**, resulting in a new **Pool B **with 122 genes (Figure 1B and Additional file 3). Surprisingly, only 20 genes in **Pool A **were also present in **Pool B **(**Pool C**, Figure. 1B). Thus, the majority of genes regulated by nandrolone at 35 days were not altered by this agent at 7 days.

### GO categories of genes altered by nandrolone

**Figure 2:**
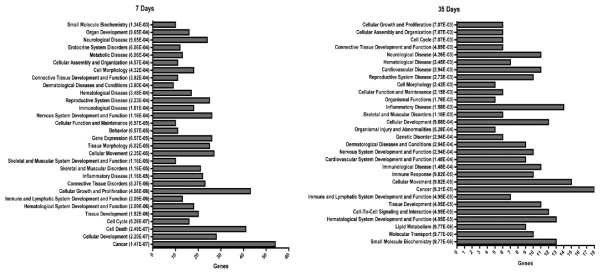
**Biological functions for nandrolone-regulated genes at 7 and 35 days**. Common groupings and biological functions of genes regulated in denervated muscle at 7 or 35 days were determined using Ingenuity Pathways analysis. Numbers in parentheses indicate the p value for enrichment of the corresponding GO biological function.

Genes regulated by nandrolone were grouped according to their designations in the gene ontology database (GO categories) to delineate common groupings and biological networks activated or suppressed in denervated muscle by nandrolone at 7 or 35 days. The biological functions of genes regulated by nandrolone at both time points are depicted in Figure 2. This analysis revealed marked differences in the biological functions of genes regulated by nandrolone at both of the time points. At 7 days, the most significant groupings were for cell cycle, cell death, cellular development and cancer, whereas at 35 days, the most significant p values were for lipid metabolism, molecular transport and small molecule biochemistry, categories that were not significantly enriched at 7 days (Figure 2). Cell-cell signaling, and cardiovascular system development and function were also enriched only at 35 days, whereas categories for gene expression, and skeletal and muscular system development, were enriched only at 7 days. Cell cycle, connective tissue development and function, skeletal and muscle disorders and cancer were enriched at both 7 and 35 days. Thus, functional groupings of genes regulated by nandrolone differed at 7 and 35 days.

### Genes altered by nandrolone at 7 or 35 days

**Figure 3:**
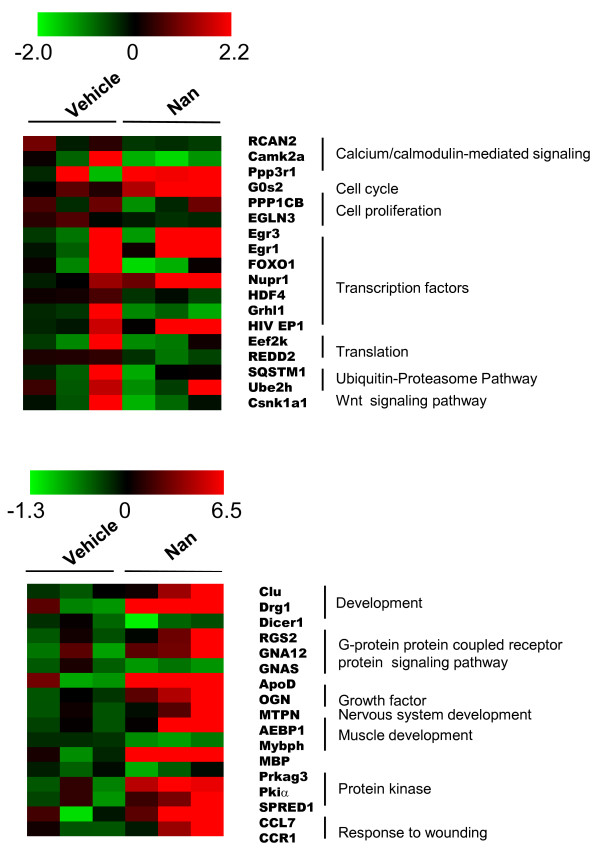
**Heat maps for selected genes regulated at 35 days**. Heat maps were generated by TM4 MultiExperiment Viewer Version 4.3.02 http://www.tm4.org using microarray expression data for selected genes from **Pool B**. *Red *represents up-regulation, while *green *represents downregulation. The color scale bar indicates Log_2 _ratio of intensities (Nan ÷ Vehicle)]. Genes are grouped by the biological functions are indicated.

Genes regulated by nandrolone at 7 days were further filtered based upon known, or potential, roles in muscle atrophy or hypertrophy, or in transcriptional regulation by the AR; the genes selected are shown in Table 1. For the purposes of comparison, the effects of nandrolone on these genes at 35 days are also shown. A similar selection process was used to identify genes of potential interest that were regulated by nandrolone at 35 days (Table 2), which, again are shown together with corresponding effects of nandrolone at 7 days. A heat map depicting normalized expression values for each individual microarray for selected genes that were significantly altered by nandrolone at 35 days is shown in Figure 3. Comparison of expression changes in Table 2 with corresponding changes for each microarray (Figure 3) revealed good agreement. Overall, the direction and relative magnitude of change was similar among the microarrays for each of the genes examined.

**Table 1:** Selected GO category and genes that are altered at 7 days denervation, and comparison with expression at 35 days.

GO category	Gene Name	Gene Symbol	7 days (Nan vs Veh)	35 days (Nan vs Veh)
Calcium/calmodulin-mediated signaling	Thrombospondin 1	THBS1	-2.27	2.55
Muscle contraction	Galanin	GAL	-1.43	10.82
	Tropomyosin 3, gamma	TPM3	-2.00	-1.12
Proteolysis	A disintegrin-like and metallopeptidse with thrombospondin type 1 motif, 1	ADAMTS1	-1.69	2.33
	Serpin peptidase inhibitor, class E, member 1	SERPINE1	-2.86	3.09
Steroid_hormone_receptor_activity	Nuclear receptor subfamily 4, group A, member 1	NR4A1	-4.00	1.75
	Nuclear receptor subfamily 4, group A, member 2	NR4A2	-1.89	2.12
	Nuclear receptor subfamily 4, group A, member 3	NR4A3	-5.88	1.28
Transcription	Activating transcription factor 3	ATF3	-5.56	2.36
	B-cell leukemia/lymphoma 6	BCL6_PREDICTED	-1.72	-1.06
	Basic helix-loop-helix domain containing, class B3	BHLHB3	1.67	-1.05
	B-cell translocation gene 2, anti-proliferative	BTG2	-2.63	1.58
	Transcription factor 4	TCF4	-1.39	1.18
	Transducin-like enhancer of split 1, homolog of Drosophila E(spl)	TLE1_PREDICTED	-1.75	-1.08
	Splicing factor, arginine/serine-rich 10 (transformer 2 homolog, Drosophila)	SFRS10	1.55	-1.09
Ubiquitin -Proteasome Pathway	Ubiquitin-conjugating enzyme E2E 2 (UBC4/5 homolog, yeast)	UBE2E2	-1.32	-1.08
	Ubiquitin specific protease 12	USP12_PREDICTED	-1.23	1.35

Abbreviation: Nandrolone, Nan; Vehicle, Veh.

**Table 2:** Selected GO category and genes that are altered at 35 days, and comparison with changes at 7 days.

GO category	Gene Name	Gene Symbol	35 days	7 days
			(Nan vs Veh)	**(Nan vs Veh**)
Calcium/calmodulin-mediated signaling	Regulator of Calcineurin 2 (Down syndrome critical region gene 1-like 1)	RCAN2	-1.69	1.26
	Calcium/calmodulin-dependent protein kinase II, alpha	Camk2a	-3.23	-1.30
	Calcineurin B, type1	Ppp3r1	2.11	1.12
Cell cycle	G0/G1 switch gene 2	G0s2	2.07	-1.45
	Transducer of ERBB2, 2	Tob2	-1.43	1.03
Cell proliferation	Protein phosphatase 1, catalytic subunit, beta isoform	PPP1CB	-1.92	1.02
	EGL nine homolog 3	EGLN3	-1.64	1.12
Development	Clusterin	Clu	2.52	1.05
	Developmentally regulated GTP binding protein 1	Drg1	3.97	1.24
	Dicer1	Dicer1	-2.00	-1.14
	Sortilin 1	SORT1	-1.82	1.50
G-protein coupled receptor protein signaling pathway	Regulator of G-protein signaling 2	RGS2	1.96	1.08
	Guanine nucleotide binding protein, alpha 12	GNA12	1.94	1.07
	GNAS complex locus	GNAS	-2.38	1.24
Growth factor	Apolipoprotein D	Apo D	14.73	3.00
	Osteoglycin	OGN	2.60	1.27
Muscle development	Myotrophin	Mtpn	1.92	1.02
	AE binding protein 1	AEBP1	3.44	2.60
Muscle contraction	Myosin binding protein H	Mybph	-1.82	1.15
Nervous system development	Myelin basic protein	MBP	12.06	2.85
Protein kinase	Protein kinase, AMP-activated, gamma 3 non-catalytic subunit	Prkag3	-1.64	1.05
	Protein kinase inhibitor, alpha	Pkiα	2.47	-1.08
	Protein kinase, AMP-activated, a1 catalytic unit	Prkaa1	1.93	-1.14
	Sprouty protein with EVH-1 domain 1, related sequence	SPRED1	2.93	1.58
Response to wounding	Chemokine (C-C motif) ligand 7	CCL7	1.70	1.05
	Chemokine (C-C motif) receptor 1	CCR1	1.55	1.23
Transcription	Early growth response 1	Egr1	2.28	-2.33
	Early growth response 2	Erg2	2.54	-3.85
	Early growth response 3	Egr3	2.29	-4.76
	Forkhead box protein O1A	FOXO1	-2.22	1.22
	Transforming, acidic coiled-coil containing protein 2	TACC2	-2.38	1.41
	Nuclear protein 1	Nupr1	2.17	1.37
	Heat shock transcription factor 4	HSF4	-1.72	1.10
	carboxy-terminal domain, RNA polymerase II, polypeptide A	Ctdsp1	-1.54	1.06
	Grainyhead-like 1	Grhl1	-2.70	1.22
	Human immunodeficiency virus type I enhancer binding protein 1	HIV EP1	1.72	1.16
	TSC22-related-inducible leucine zipper protein 2	TSC22	-1.19	2.30
Translation	Eukaryotic elongation factor-2 kinase	Eef2k	-2.44	1.24
	DNA-damage-inducible transcript 4-like	REDD2/DDIT4L	-1.72	-1.32
Ubiquitin -Proteasome Pathway	Sequestosome 1	SQSTM1	-1.92	1.37
	UBIQUITIN-CONJUGATING ENZYME E2H	Ube2h	1.97	1.12
Wnt signaling pathway	Casein kinase 1, alpha 1	Csnk1a1	-2.44	-1.37

Abbreviations: Nandrolone, Nan; Vehicle, Veh.

#### Effects of nandrolone on gene expression by biological function

##### Translation

At 35 days, nandrolone reduced expression of two inhibitors of translation, REDD2, and Eef2 kinase (Table 2). At 7 days, nandrolone did not significantly alter expression of either gene.

##### Development and Muscle Development

Nandrolone altered expression of genes in development at 35 days by 2- to 5-fold. It upregulated clusterin (Clu) and developmentally regulated GTP-binding protein 1 (Drg1), and downregulated Dicer1 and sortilin 1 (SORT1) (Table 2). At 7 days, nandrolone increased SORT1 expression, but did not significantly alter expression of Clu, Drg1, or Dicer1 (Table 2). At 35 days, nandrolone induced expression of two genes linked to muscle development, myotrophin (Mtpn), a growth factor, and AE binding protein 1 (AEBP1), a transcription factor (Table 2). At 7 days, myotrophin expression was unaffected by nandrolone while AEBP1 expression was increased, but the magnitude of the change was three quarters of that stimulated by nandrolone at 35 days (Table 2). At 7 days, nandrolone reduced expression of one gene linked to muscle development, Cmya1_predicted (Additional file 1).

##### Calcium/calmodulin-mediated signaling

Several genes encoding molecules involved in calcium/calmodulin-mediated signaling were differentially altered at 35 days as compared to 7 days. Regulator of calcineurin 2 (RCAN2/DSCR1L1) was significantly downregulated by nandrolone at 35 days but was upregulated at 7 days (Table 2). Thrombospondin 1 (THBS1) was upregulated by nandrolone at 35 days but down-regulated at 7 days (Table 1). Calcineurin B, type1 (Ppp3r1) was up regulated by nandrolone at 35 days but unchanged at 7 days (Table 2).

##### Growth factors and response to wounding

Nandrolone altered the expression of several growth factors. At 35 days, nandrolone markedly upregulated apolipoprotein-D (Apo D, Table 2) and galanin (GAL, Table 1). At 35 days, nandrolone also upregulated osteoglycin, chemokine (C-C motif) ligand 7 and chemokine (C-C motif) receptor 1 (Table 2) and the Wnt inhibitors secreted frizzled-related peptides 2 and 4 (Additional file 3). Expression of these genes was not affected by nandrolone at 7 days, with the exception of galanin (Table 1). At 7 days, nandrolone upregulated osteomodulin, adiponectin-C1q and collagen domain containing, and Sema3b (Additional file 1). At 7 days, nandrolone downregulated sclerostin domain containing 1 (Additional file 1), a BMP-1 antagonist [29].

##### Protein kinases and their regulators

Genes encoding or regulating protein kinases were also differentially regulated by nandrolone at both 7 and 35 days. At 35 days, nandrolone upregulated the following: protein kinase inhibitor alpha (Pkiα); the a1 catalytic subunit of AMP-activated protein kinase (Prkaa1); and Sprouty protein with EVH-1 domain 1 related sequence (SPRED1) (Table 2). At 35 days, nandrolone downregulated the gamma 3 non-catalytic subunit of AMP-activated protein kinase (Prkag3), and calcium/calmodulin-dependent protein kinase II, alpha (Camk2a) (Table 2). At 7 days, nandrolone upregulated SPRED1, although to a lesser extent than at 35 days, but did not alter expression of Pkiα, Prkag3, or Camk2a (Table 2). At 7 days, nandrolone downregulated tribbles homolog 1, a modulator of MAPK pathways (Additional file 1).

##### Transcription/RNA processing

At 35 days, nandrolone upregulated selected transcription factors by 1.5- to 2.8-fold, including early response genes (Egr1, Egr2, and Egr3), the human immunodeficiency virus type I enhancer binding protein 1 (HIV EP1), and Nupr1, a tumor suppressor that regulates transcription and has been associated with cardiac muscle hypertrophy [30] (Table 2). Nandrolone also upregulated ATF3 at 35 days (Table 1). At 35 days, nandrolone repressed forkhead box protein O1A, more commonly referred to as FOXO1, the designation used hereafter. Also repressed by nandrolone at 35 days were transforming, acidic coiled-coil containing protein 2 (TACC2), and heat shock transcription factor 4 (HSF4) (Table 2). Genes for three transcriptional coregulators were repressed by nandrolone at 35 days: grainyhead like (Grhl1), AT hook, and carboxyterminal domain RNA polymerase II small phosphatase (Ctdsp1) (Table 2 and Additional file 3).

A striking aspect of the genes altered at 7 days was that the 6 genes most greatly downregulated by nandrolone were transcription factors (range -4.8 fold for Egr3 to -2.33 for Egr1) (Table 2). These included the early response factors Egr1-3 (Table 2), as well as Ier2 and Ier5 (Additional file 1), some of which were upregulated by nandrolone at 35 days (Egr1-3) (Table 2). At 7 days, nandrolone repressed three orphan nuclear receptors (NR4A1, NR4A2 and NR4A3) (Table 1) and several transcriptional coregulators: Ankrd1, one of a family of molecules that transmits signals from the contractile apparatus to the nucleus [31] (Additional file 1), and TLE1_PREDICTED, a transcriptional corepressor (Table 1). Also repressed were BTG2, an antiproliferative factor which regulates transcription in a p53-dependent manner, and BCL6_PREDICTED, a transcriptional repressor involved in morphogenesis (Table 1).

At 35 days, nandrolone altered expression of genes involved in RNA processing. Notable among these was Dicer 1, as noted above (Table 2). At 7 days, nandrolone reduced expression of DEAH (Asp-Glu-Ala-His) box polypeptide 36, an RNA helicase (Additional file 1).

##### Other pathways

At 35, but not 7 days, nandrolone upregulated expression of ubiquitin-conjugating enzyme E2H (Ube2h) and down-regulated sequestosome 1 (SQSTM1) (Table 2). Nandrolone downregulated ring finger protein (C3H2C3) 6, an SCF type ubiquitin ligase, at 35 but not 7 days (Additional file 3). Nandrolone also down-regulated the Wnt-signaling molecule casein kinase 1, alpha 1 (Csnk1a1) (Table 2) at 35 days.

##### Validation of cDNA microarray results with qPCR

**Figure 4:**
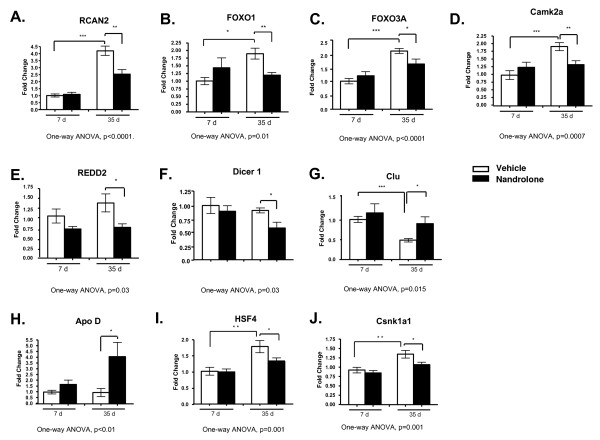
**Real time PCR quantification of mRNA levels of selected genes altered by nandrolone**. **A-J**. Levels of mRNA for RCAN2, FOXO1, FOXO3A, REDD2, Dicer 1, HSF1, Clu, Apo D, Csnk1a1 and CaMK2a are shown. Data are presented as means ± SEM. Groups contained 7 to 8 animals. Data were analyzed by one-way ANOVA; p values for the F statistic are shown under each panel. *p < 0.05, **p < 0.01, ***p < 0.001 *versus *the indicated group.

To confirm selected microarray results, and to examine biological variability among equivalently treated animals, we examined mRNA levels in denervated gastrocnemius muscles using real time PCR. In this analysis, we also included FOXO3A, which is closely related to FOXO1 and exerts similar effects on the expression of MAFbx [13]. There was good agreement between the microarray and qPCR data as far as direction and relative magnitude of changes in gene expression. At 35 days, nandrolone significantly increased expression of ApoD and Clu and decreased expression of REDD2, RCAN2, FOXO1, Dicer 1, CamK2a, Csnk1a1 and HSF4 (Figure 4). At 7 days, effects of nandrolone on gene expression were: in the opposite direction for FOXO1, FOXO3A and Camk2a; smaller for REDD2, Clu and, ApoD; and minimal for RCAN2, Csnk1a1 and HSF4 (Figure 4).

##### Western blotting

**Figure 5:**
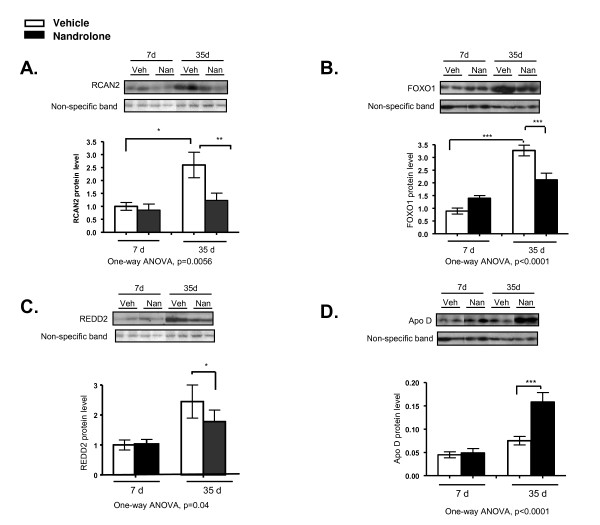
**Western blotting analysis of protein levels for selected gene products**. **A-D**. Western blotting analysis of the effects of nandrolone on levels of RCAN2, FOXO1, REDD2, and ApoD proteins in denervated gastrocnemius muscle are shown. Groups contained data for 7 to 8 animals each. Data are presented as means ± SEM and were analyzed by one-way ANOVA; p values for the F statistic are shown under each panel. *, p < 0.05, **, p < 0.01, ***, p < 0.001 *versus *the indicated group.

Effects of nandrolone on levels of selected proteins in denervated gastrocnemius muscle were assessed by Western blotting. These effects agreed well with changes in mRNA levels (Figures 4 and 5). Nandrolone significantly reduced levels of RCAN2, FOXO1 and REDD2 at 35 but not 7 days, and significantly increased ApoD levels at 35 days but not 7 days (Figure 5).

### Changes over time in gene expression in denervated muscle

#### Microarray data

Gene expression in denervated muscle from vehicle-treated rats was compared at 7 and 35 days. A complete listing of the genes for which expression was found to change significantly between 7 and 35 days is shown in Additional file 2. This list included 318 unique genes involved in cell growth and proliferation, connective tissue development and function, cell cycle, and cell death.

Genes demonstrating altered expression included those expressing kinases, phosphatases and transcriptional regulators but not growth factors. When comparing expression levels at 35 versus 7 days for transcriptional coregulators, CREBBP, RNPC2, PRRX1, Nrip1 and NMI were upregulated by 2- to 3.98-fold while Tgif, Lmcd1, Ankrd1 and Ankrd2 were downregulated from -2.75-to -23.81-fold (Table 3). There were 24 other transcriptional regulators for which expression changed between 7 and 35 days, with alterations in expression ranging from a 3.28-fold increase to -6.85-fold decrease (Table 3). The largest increases in expression were for TSC22D4, BHLHB3, and DBP, while the greatest decreases in expression were observed for BTG2, Egr2, and RCAN1 (Table 3).

**Table 3:** Selected GO category and genes that are altered at 35 days denervation, in comparison with 7 days denervation

GO category	Gene Name	Gene Symbol	Fold-Change (35 vs 7)
**Transcriptional Coregulators**	creb binding protein	CREBBP	3.98
	rna-binding region (rnp1, rrm) containing 2	RNPC2	2.85
	paired related homeobox 1	PRRX1	2.40
	nuclear receptor interacting protein 1 (predicted)	Nrip1	2.06
	n-myc (and stat) interactor	NMI	2.00
	homeodomain interacting protein kinase 2 (predicted)	Hipk2	-2.54
	tg interacting factor	Tgif	-2.75
	lim and cysteine-rich domains 1 (predicted)	Lmcd1	-4.27
	Ankyrin repeat domain 1 (cardiac muscle)	Ankrd1	-4.48
	Ankyrin repeat domain 2 (stretch responsive muscle)	Ankrd2	-23.81
**Transcription Factors and Other Transcriptional Regulators**			
	TSC22 domain family, member 4	TSC22D4	3.28
	basic helix-loop-helix family, member e41	BHLHB3	3.17
	D site of albumin promoter (albumin D-box) binding protein	DBP	3.07
	activating transcription factor 5	ATF5	2.53
	MAX gene associated	MGA (includes EG:23269)	2.40
	myeloid/lymphoid or mixed-lineage leukemia (trithorax homolog, Drosophila)	MLL	2.38
	POZ (BTB) and AT hook containing zinc finger 1	PATZ1	2.34
	Kruppel-like factor 5 (intestinal)	KLF5	2.25
	thyrotrophic embryonic factor	TEF	2.25
	single-minded homolog 2 (Drosophila)	SIM2	2.16
	TSC22 domain family, member 3	TSC22D3	2.10
	myogenic differentiation 1	MYOD1	2.10
	Kruppel-like factor 15	KLF15	2.05
	CCAAT/enhancer binding protein (C/EBP), delta	CEBPD	2.05
	polycomb group ring finger 6	PCGF6	-2.08
	SRY (sex determining region Y)-box 4	SOX4	-2.13
	v-maf musculoaponeurotic fibrosarcoma oncogene homolog F (avian)	MAFF	-2.33
	zinc finger protein 367	ZNF367	-2.67
	Ankyrin repeat and SOCS box-containing 5	ASB5	-2.95
	aryl hydrocarbon receptor nuclear translocator-like	ARNTL	-3.07
	LIM and cysteine-rich domains 1	LMCD1	-4.27
	regulator of calcineurin 1	RCAN1	-5.05
	BTG family, member 2	BTG2	-5.35
	early growth response 2 (Krox-20 homolog, Drosophila)	Egr2	-6.85
			
**Kinases**	v-erb-b2 erythroblastic leukemia viral oncogene homolog 2, neuro/glioblastoma derived oncogene homolog (avian)	ERBB2	4.08
	neurotrophic tyrosine kinase, receptor, type 2	NTRK2	3.33
	phosphoinositide-3-kinase, class 2, beta polypeptide	PIK3C2B	2.50
	cytidine monophosphate (UMP-CMP) kinase 2, mitochondrial	CMPK2	2.43
	discoidin domain receptor tyrosine kinase 2	DDR2	2.33
	activin A receptor, type IIB	ACVR2B	2.28
	protein kinase, cAMP-dependent, regulatory, type II, beta	PRKAR2B	2.08
	EPH receptor B3	EPHB3	1.94
	cell division cycle 2-like 6 (CDK8-like)	CDC2L6	1.93
	protein kinase, AMP-activated, beta 2 non-catalytic subunit	PRKAB2	1.89
	PTEN induced putative kinase 1	PINK1	1.81
	STE20-related kinase adaptor beta	STRADB	-1.87
	mex-3 homolog B (C. elegans)	MEX3B	-1.92
	RIO kinase 3 (yeast)	RIOK3	-2.32
	tribbles homolog 1 (Drosophila)	TRIB1	-2.45
	uridine-cytidine kinase 2	UCK2	-2.56
	membrane protein, palmitoylated 6 (MAGUK p55 subfamily member 6)	MPP6	-2.84
			
**Phosphatases**	protein tyrosine phosphatase, receptor type, D	PTPRD	6.96
	dual specificity phosphatase 11 (RNA/RNP complex 1-interacting)	DUSP11	-1.54
	protein phosphatase 1, regulatory (inhibitor) subunit 3C	PPP1R3C	-1.84
	protein phosphatase 1J (PP2C domain containing)	PPM1J	-1.91
	protein tyrosine phosphatase, receptor type, C	PTPRC	-2.21
	protein tyrosine phosphatase-like (proline instead of catalytic arginine), member A	PTPLA	-2.95
	nudix (nucleoside diphosphate linked moiety X)-type motif 4	NUDT4	-3.62

Seventeen kinases demonstrated significant changes in expression ranging from a 4.08-fold increase to a -2.84-fold decrease. Kinases with the most highly increased expression included ERBB2, NTRK2, and PIK3C2B, while those with the greatest decrease in expression included MPP6, TRIB1, and UCK2 (Table 3). Seven phosphatases demonstrated altered expression, with 6 being decreased by -1.54- to -3.62-fold and one being increased by 6.96-fold (protein tyrosine phosphatase, receptor type D, PTPRD) (Table 3).

#### Verification of selected microarray data by real time PCR

**Figure 6:**
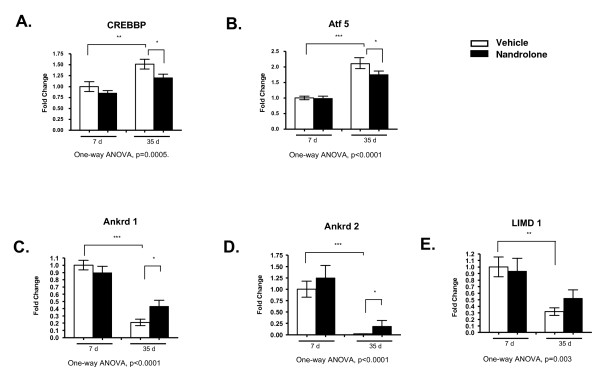
**Real time PCR quantification of mRNA levels of selected transcriptional regulators differentially regulated at 7 versus 35 days**. **A-E**, levels of mRNA for CREBBP, Atf5, Ankrd 1, Ankrd 2 and LIMD 1 are shown. Groups contained data for 7 to 8 animals each. Data are means ± SEM and were analyzed by one-way ANOVA; p values for the F statistic are shown under each panel. *, p < 0.05, **, p < 0.01, ***, p < 0.001 *versus *the indicated group.

The results of the microarray analysis were confirmed for selected genes by real time PCR (Figure 6). A comparison of findings from microarray and real time PCR analysis revealed that the direction and magnitude of change in expression were similar. As compared to 7 days, expression at 35 days was significantly different for the transcriptional coregulators Ankrd1, Ankrd2, and CREBBP, as well as for the transcription factors Atf5 and LIMCD 1.

### Correlation between gene expression changes and nandrolone response

**Figure 7:**
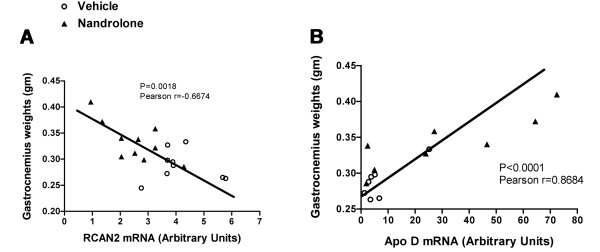
**Correlations for selected genes with gastrocnemius size and RCAN2 mRNA levels**. **A**. Correlation at 35 days between gastrocnemius muscle weight and mRNA levels for RCAN2. Each data point represents the muscle weight and RCAN2 mRNA level for a single animal. **B**. As in a. except that ApoD mRNA levels are shown.

To gain insights into physiological significance of gene expression changes, we analyzed the relationship between gastrocnemius muscle size at 35 days and magnitude of gene expression change induced by nandrolone. For this analysis we chose the two genes for which nandrolone had the largest effect on mRNA levels as determined by real time PCR: RCAN2 and ApoD. There was a significant negative correlation between RCAN2 mRNA levels and gastrocnemius muscle weight (Figure 7A). A positive correlation was observed between ApoD mRNA and weights of denervated gastrocnemius (Figure 7B).

## Discussion

### Nandrolone effects on gene expression over time

This study sought insights into the molecular basis for the observation that administration of nandrolone for 7 days slowed denervation atrophy when begun at day 29 after nerve transection (35 days), but had no effect on atrophy when initiated at the time the nerve was severed (7 days). The findings indicated that nandrolone regulated an almost entirely different set of genes at 7 days compared to 35 days. A marked change in the expression in denervated muscle of genes involved in the control of transcription and intracellular signaling was observed between 7 and 35 days.

Among genes regulated by nandrolone at 35 but not 7 days were molecules that drive muscle atrophy (FOXO1), inhibit protein synthesis (REDD2), regulate calcineurin (RCAN2), and determine Wnt signaling (Csnk1a1). FOXO1 is a transcription factor known to reduce muscle size [32,33], and to upregulate MAFbx and MuRF1 and the protein synthesis inhibitor 4EBP1 [13,14,32,34]. Nandrolone-induced reductions in FOXO1 at 35 but not 7 days agree well with the prior observation that nandrolone reduced expression of MAFbx and MuRF1 at 35 but not 7 days [27]. The findings suggest that downregulation of FOXO1 represents a likely mechanism by which nandrolone slows denervation atrophy.

To our knowledge, neither RCAN2 nor calcineurin have been previously suggested to be involved in nandrolone action or denervation atrophy. Calcineurin is a calcium-calmodulin-dependent dual-specificity phosphatase which promotes the slow-twitch endurance muscle fiber type [35]. At 35 days, nandrolone reduced expression of RCAN2, and RCAN2 levels were inversely correlated with the size of denervated gastrocnemius. Nandrolone also altered the expression of a regulatory subunit of calcineurin, calcineurin B, type 1. Of interest, in studies of calcineurin function in the pathogenesis of cardiac hypertrophy, calcineurin activity has been shown to be reduced by MAFbx, FOXO1 or FOXO3A, and to be directly linked to myocardiocyte size [17,36]. The role of calcineurin in hypertrophy of normal skeletal muscle hypertrophy, or spontaneous recovery from muscle atrophy, is controversial [18,35,37-40]. Its roles in denervated muscle, or androgen action, are unknown. We are now investigating the relationship between nandrolone action and calcineurin in atrophied skeletal muscle.

Increased expression of inhibitors of mTOR, such as REDD1 and REDD2, have been linked to decreases in cell size and protein synthesis and have been suggested to promote muscle atrophy [20,21,26,41]. mTOR is a master regulator of protein synthesis [26,41] and is necessary for muscle hypertrophy and recovery of muscle size after muscle atrophy [18]. Upregulation of mTOR inhibitors has been described during muscle atrophy caused by glucocorticoids [20] or alcohol ingestion [21] and has been implicated in mTOR inhibition due to glucocorticoids in cultured myoblasts [20]. Of interest, testosterone prevented upregulation of REDD1 in dexamethasone-treated rats and cultured myoblasts and normalized mTOR activity in cultured cells exposed to dexamethasone [42]. Consistent with these findings, in denervated muscle, nandrolone reduced REDD2 mRNA and protein at 35 but not 7 days, suggesting that reduction in expression of this protein, and subsequent increases in mTOR activity, may represent one mechanism by which nandrolone slows denervation atrophy.

Other genes upregulated at 35 days by nandrolone include Wnt signaling molecules (e.g., Csnk1a1, sFRP2 and sFRP4) and ApoD. Wnt signaling appears to be important to muscle hypertrophy [43,44]. Of interest, in a cell culture system, the intracellular target of Wnt signaling, β-catenin, can be activated by androgens through physical interactions between β-catenin and the AR [45]. Upregulation of ApoD, a lipoprotein believed to participate in uptake or intercellular transport of ligands [46], correlated well with denervated muscle size at 35 days. Upregulation of this gene has also been observed in muscle hypertrophy [47]. The significance of these changes in ApoD expression is unknown.

### Molecular determinants of nandrolone-induced alterations in gene expression

An additional objective of this study was to examine the possibility that changes over time in gene expression could provide insights into the molecular determinants for the marked time-dependent effects of nandrolone on gene expression in denervated muscle. These time-dependent effects were dramatically demonstrated by the minimal overlap of genes regulated at 7 versus 35 days, despite the fact that over 100 genes were regulated by this agent at each time point. Equally interesting was the finding that the list of genes regulated by nandrolone at 35 but not 7 days included several shown to be critical to muscle atrophy, specifically FOXO1 (this study), and MAFbx and MuRF1 [27].

These time-dependent actions of nandrolone occurred on a background of changes over time in expression of over 300 genes in denervated muscle, that included many genes that function in intracellular signaling and transcriptional regulation, such as kinases, phosphatases, transcription factors and transcriptional coregulators. The AR is a transcription factor, and the classical mechanism by which drugs such as nandrolone signal through the AR is transcriptional regulation by the AR when bound to chromatin, or to other transcription factors. Transcriptional activity of the AR is dependent upon binding of coregulators [48,49], and interactions with nearby transcription factors. Coregulators modify chromatin structure to repress or transactivate specific genes, and their binding to AR is critical to its transcriptional control of target genes [48,49].

Interactions between the AR and other transcription factors form one basis for transcriptional repression (e.g., [50] and references cited therein) and can determine whether a steroid hormone receptor, such as the AR, is able to transactivate specific genes [51]. Interdependence of AR actions and levels of specific transcription factors were illustrated by findings that gene knockdown with and siRNA against Oct-1 abrogated repression of MAFbx by testosterone [50]. The concept that levels of a transcriptional regulator can profoundly affect transcriptional programs was demonstrated by the effects of PGC-1α on muscle fiber type and mitochondrial biogenesis ([52] and references therein). Thus, one model that would explain the time-dependent effect of nandrolone is variation over time in levels or activity of transcription factors and/or coregulators with which the AR interacts at target genes.

Marked changes in the expression of several transcriptional coregulators were observed between days 7 and 35 after denervation, with the most dramatic being the large reductions in expression levels for Ankrd1 and Ankrd2. The influence of these coregulators on transcriptional regulation by the AR has not been described. Among coregulators with less dramatic changes in expression between 7 and 35 days, several are known to regulate transcriptional activities of the AR: CREBBP [53], Tgif [54], Ctdsp1[55] and NRIP1 [56].

Interactions between the AR and other transcription factors have been reported for GR, Ets1, Oct1, NFkB, FOXO1 and AP1 [57-63]. Many transcription factors demonstrated altered expression between 7 and 35 days. Although interactions of these transcription factors with the AR have not been reported, it remains possible that these occur, and that changes in their expression may be linked to some of the time dependent effects of nandrolone.

Other mechanisms may also be involved in, or be critical to, time-dependent effects of nandrolone on gene expression. These may include changes in phosphorylation status of transcriptional regulators, or non-genomic effects of nandrolone mediated through interactions with kinases (e.g., ERBB2, PI3KC2B), G-proteins, or other intracellular signaling molecules [64]. For example, it has been demonstrated that transcriptional activity of PGC-1α is determined by activity of the kinases AMPK and p38 MAPK [65,66].

Thus, the findings suggest several possible mechanisms that may explain the time-dependent effects of nandrolone on gene expression in denervated muscle. Future investigations focused on more detailed time course studies, interactions of proteins encoded by these regulatory genes with the AR, and their effects on nandrolone target genes such as FOXO1 or MAFbx, hold the promise of identifying the specific molecular interactions by which nandrolone exerts such profoundly different actions over time.

### Comparison with other studies of androgen actions in atrophied muscle

An interesting consideration is that the effects of nandrolone to slow atrophy of denervated gastrocnemius are much greater than its effects to increase the mass of normal rat muscles, including gastrocnemius, [67] but that both of these actions of nandrolone are considerably smaller than the dramatic effect of androgens to increase the size of the rat levator ani muscle [68,69]. It is possible that similar mechanisms determine androgen responsiveness of these normal and denervated muscles. It is also possible that the marked changes in expression of key regulatory molecules that occurs with time after denervation play important roles in determining androgen sensitivity of denervated muscle that are distinct from those that specify the androgen responses of normal muscle and the levator ani.

In either case, the time-dependent differences in nandrolone effects on denervated muscle appear to be one manifestation of a more general influence of the physiological state of skeletal muscle on responses to androgens. For example, genes regulated by nandrolone at 7 or 35 days differed from those regulated by androgens in other genomic studies. In agreement with our findings in denervated muscle at 35 days, in HIV-infected men, testosterone altered the expression of genes regulating IGF-1 signaling, muscle development, transcription, cell cycle and apoptosis, and Wnt signaling, but in contrast to denervated muscle, not those involved in calcineurin signaling or translation [70]. In dexamethasone-treated rats, testosterone reversed dexamethasone-induced changes in expression of FOXO1, but also reduced expression of REDD1 and 4EBP1 [42], genes that were not found to be nandrolone responsive in denervated muscle. In AR-deficient mice, alterations in gene expression were noted for genes for myoblast differentiation and polyamine synthesis, as well as those for cell cycle progression and Wnt signaling [71]. Loss of the AR also affected expression of IGF-II and several other growth factors, but, not genes regulating calcineurin signaling or translation [71].

## Conclusions

Genes regulated by nandrolone in denervated muscle at 7 days were almost entirely different from those regulated by this agent to 35 days. These time-dependent nandrolone effects were associated with many changes in expression in denervated muscle of genes involved in the control of transcription and intracellular signaling. Genes regulated by nandrolone at 35 but not 7 days include factors known to drive muscle atrophy (FOXO1), inhibit protein synthesis (REDD2), regulate calcineurin (RCAN2), and determine Wnt signaling. Marked changes in expression of transcriptional regulators known or suspected to interact with the AR occur between 7 and 35 days, and their differential regulation may explain the time-dependence of nandrolone effects on gene expression in denervated muscle, at least in part.

## Methods

### Animals, sciatic nerve transection and drug administration

The analysis used gastrocnemius muscle from male Wistar-Hannover rats that had undergone left sciatic nerve transection followed by the administration of nandrolone or vehicle beginning either on the day of surgery or 29 days thereafter. Effects of nandrolone on gastrocnemius weights and mRNA levels for MAFbx and MuRF1 have been previously reported [27]. A summary of the experimental design is presented in Figure 1A. Animals had been sacrificed 7 days after starting nandrolone or vehicle (e.g, at days 7 or 35). Muscle tissue was flash-frozen at sacrifice and stored at -80 ˚C. The animal studies were reviewed and approved by the Institutional Animal Care and Use Committee of the James J. Peters VA Medical Center.

### Microarray analysis

Microarray analysis was performed for total RNA from 12 animals, 3 each from: day 7 vehicle, day 7 nandrolone, day 35 vehicle, and day 35 nandrolone; one microarray was used for each animal. Total RNA from gastrocnemius muscles from the denervated hind limb was used for these studies. Muscle (20 mg) was homogenized in guanidinium isothiocyanate buffer on ice using a Polytron. RNA was extracted using phenol-chloroform [72], then further enriched using RNAeasy minicolumns (Qiagen, Valencia, CA). Verification of RNA integrity and microarray analysis were performed by the Microarray Core Facility at the Mount Sinai School of Medicine. Samples selected for microarray analysis yielded RNA integrities greater than 8.0 using an Agilent Bioanalyzer. Microarray analysis employed Affymetrix rat genome 230 2 arrays and was performed using the recommended procedures of the manufacturer. Microarray data has been deposited in NIH GEO (Accession number GSE17959).

### Data filtering and mining

Differentially expressed genes were identified using BRB Array Tools version 3.6.2, developed by Dr. Richard Simon and Amy Peng Lam. Data from the Affymetrix plate reader was loaded directly into the software. Affymetrix Present/Absent calls were not included in the analysis. Predefined BRB Array Tools software settings were used for normalization and filtering. Data for each array were normalized using the median for the entire array. Expression values were set to 10 when they were below this value. Expression values were excluded unless the values for at least 20% of the arrays were 1.5-fold or more different from the median for that probe set. The significance of differences in expression among groups was determined using an F-test, with significance set at p < 0.05. Because the number of genes modulated by nandrolone at each time point was not very large, all probes yielding a significant difference at p < 0.05 were included in subsequent analysis. For the comparison of gene expression in denervated muscle at 7 and 35 days, a much larger number of genes was identified; to limit the list of candidates somewhat, only those differing at p < 0.01 were included in subsequent analysis.

Values for fold-change (e.g., nandrolone versus vehicle at 7 or 35 days) were calculated using geometric means. Biological functions of differentially expressed genes were determined using Ingenuity Pathways, NIH DAVID [73,74] and GeneCards at http://www.genecards.org[75]. Subsets of genes regulated by nandrolone at 7 or 35 days were selected for additional analysis based upon known or proposed relationships to muscle atrophy and hypertrophy, or transcriptional regulation by androgens.

Heat maps were generated using the microarray expression data that had been normalized relative to the mean for all expression values for the array and were generated using TM4 MultiExperiment Viewer Version 4.3.02 http://www.tm4.org. Fold change for the expression value for each gene and microarray was calculated relative to the arithmetic mean for the vehicle group for that gene.

Tests for enrichment of biological themes were performed using Ingenuity Pathways Analysis (Ingenuity systems, http://www.ingenuity.com).

### Quantitative real time PCR (qPCR)

Total RNA (1 μg) was used to prepare cDNA libraries by reverse transcription (High Capacity cDNA Archive Kit; Applied Biosystems, Foster City, CA). Real time PCR was performed in triplicate, and the mean for the crossing points of triplicates was used in subsequent calculations. Data were normalized relative to 18S RNA [76,77]. Levels of gene expression were expressed as fold-change relative to denervated muscle from animals that were administered vehicle and sacrificed at 7 days using the 2^-ΔΔCt ^method [78]. Data are shown as mean ± SEM.

### Western blotting

Gastrocnemius (20 mg) was homogenized in 200 μl of ice-cold lysis buffer (Cell Signaling) containing protease and phosphatase inhibitors (Roche) using a Polytron. Homogenates were cleared by centrifugation at 4°C in a microcentrifuge. Proteins were denatured by boiling in SDS-PAGE sample buffer containing β-mercaptoethanol and resolved by SDS-PAGE. Proteins were then electrophoretically transferred to PVDF membranes and probed with appropriate antibody. In these studies, immunoreactive bands were visualized by chemiluminesence using ECL plus western-blotting detection system (GE healthcare) and captured on photographic film with subsequent digitization of images using a scanner. Intensity of bands on digitized images was quantified using Imagequant TL (GE Life Sciences, Piscataway, NJ). Choice of a protein for normalization was challenging for these studies because long-term denervation resulted in profound alterations in levels of many cellular proteins, which was readily appreciated on Coomassie Blue-stained SDS-page gels of skeletal muscle lysates. Commonly used housekeeping genes such as β-tubulin, α-actin and GAPDH proved to be unreliable for animals with prolonged denervation. Therefore, we have used the intensity of a neighboring non-specific band for normalization. Antibodies that were used in these studies include: RCAN2 (mouse polyclonal anti-DSCR1L1, Novus Biologicals, Littleton, CO, 1:500 dilution), FOXO1 (L27) (Cell signaling, Danvers, MA, 1:1,000 dilution), REDD2 (Abcam, Cambridge, MA, 1:500 dilution), Apo D (Abcam, 1:1,000 dilution), and anti-β-tubulin (Abcam, 1:2,000 dilution). Data are shown as mean ± SEM, and differences among means were determined by ANOVA as above.

### Statistics

For real time PCR and western blotting data, differences among means were determined using one way ANOVA with a Newman-Keuls multiple comparison test post-hoc to test for significance of differences between pairs of means. Linear regression analysis was used to test correlations. Calculations were performed using Graphad Prism 4.0c (Graph Pad Software, San Diego, CA).

## Authors' contributions

W.Q. performed part of the work, interpreted data and wrote part of the manuscript.

J.P. performed part of the work. W.A.B. wrote part of the manuscript. C.P.C. conceived the project and wrote part of the manuscript. All authors read and approved the final manuscript.

## Supplementary Material

Additional file 1 **Genes changed by nandrolone at 7 days (Pool A)**. The file lists genes for which expression was altered by nandrolone at 7 days (**Pool A**), as well as a description of each gene, its gene symbol, the name of probe set, and how much its expression changed. Click here for file

Additional file 2 **Genes with altered expression at 35 versus 7 days after denervation (Pool E)**. The file lists genes for which expression was different at 35 versus 7 days after denervation, as well as a description of each gene, its gene symbol, the name of probe set, and how much its expression changed. Click here for file

Additional file 3 **Genes altered by nandrolone at 35 days (Pool B)**. The file lists genes for which expression was altered by nandrolone at 35 days as well as a description of each gene, its gene symbol, the name of probe set, and how much its expression changed. Click here for file
