# Supplementary material for: Differential alterations in gene expression profiles contribute to time-dependent effects of nandrolone to prevent denervation atrophy
Source: BMC Genomics. 2010 Oct 22;11:596. doi: 10.1186/1471-2164-11-596 (PMC3091741; doi:10.1186/1471-2164-11-596)
Supplement: Additional file 1 — Genes changed by nandrolone at 7 days (Pool A). The file lists genes for which expression was altered by nandrolone at 7 days (Pool A), as well as a description of each gene, its gene symbol, the name of probe set, and how much its expression changed. [file 1471-2164-11-596-S1.PDF]

Additional File 1. Genes changed by nandrolone at 7 days (Pool A)

Description	Gene symbol	Probe set	Ratio of geom means
A disintegrin-like and metallopeptidse (reprolysin type) with thrombospondin type 1 motif, 1	Adamts1	1368223_at	-1.69
Actin-binding LIM protein 1 (predicted)	Ablim1_predicted	1378866_at	1.44
Activating transcription factor 3 /// activating transcription factor 3	Atf3	1369268_at	-4.55
Activating transcription factor 5	Atf5	1372601_at	1.36
Activity regulated cytoskeletal-associated protein	Arc	1387068_at	-1.64
Adaptor-related protein complex 3, mu 2 subunit	Ap3m2	1368246_at	1.34
Adenosine deaminase, RNA-specific, B1	Adarb1	1398370_at	1.37
Adiponectin, C1Q and collagen domain containing	Adipoq	1381997_at	1.62
AHNAK nucleoprotein (desmoyokin)	Ahnak	1371703_at	1.26
Alcohol dehydrogenase 1 (class I)	Adh1	1368021_at	1.36
Alpha-kinase 1 (predicted)	Alpk1_predicted	1382746_s_at	1.26
Ankyrin repeat domain 1 (cardiac muscle)	Ankrd1	1391753_at	-1.59
ATP synthase, H+ transporting, mitochondrial F1 complex, beta polypeptide	Atp5b	1380070_at	1.42
Bardet-Biedl syndrome 2 homolog (human)	Bbs2	1368509_at	1.38
Basic helix-loop-helix domain containing, class B3	Bhlhb3	1368511_at	1.38
B-cell leukemia/lymphoma 6 (predicted)	Bcl6_predicted	1379368_at	-1.72
B-cell translocation gene 2, anti-proliferative	Btg2	1386995_at	-2.63
Carboxylesterase 3	Ces3	1370363_at	1.75
Carboxypeptidase A3	Cpa3	1371151_at	1.58
Carboxypeptidase X 2 (M14 family) (predicted)	Cpxm2_predicted	1373148_at	2.21
Cardiomyopathy associated 1 (predicted)	Cmya1_predicted	1389119_at	-1.59
CCAAT/enhancer binding protein (C/EBP), delta	Cebpd	1387343_at	-1.30
Ceruloplasmin	Cp	1368419_at	1.21
Chaperonin subunit 6a (zeta)	Cct6a	1377006_at	-1.59
Chemokine (C-C motif) ligand 21b (serine)	Ccl21b	1378015_at	2.03
Chemokine (C-X-C motif) receptor 4	Cxcr4	1373661_a_at	1.64
Chondroadherin	Chad	1368788_at	1.95
Chordin-like 2 (predicted)	Chrdl2_predicted	1393954_at	-1.39
Complement component 7	C7 /// Tubb2c	1388557_at	1.48
Complement factor B	Cfb	1389470_at	1.58
Complement factor D (adipsin)	Cfd	1388602_at	2.09
C-type lectin domain family 11, member a	Clec11a	1392672_at	1.71
Cyclin D1 /// cyclin D1	Ccnd1	1371643_at	1.57
Cyclin-dependent kinase inhibitor 1A	Cdkn1a	1388674_at	1.79
Cytosolic ovarian carcinoma antigen 1 (predicted)	Cova1_predicted	1384198_at	-1.33
Cytotoxic granule-associated RNA binding protein 1	Tia1	1374786_at	1.24
DEAH (Asp-Glu-Ala-His) box polypeptide 36	Dhx36_predicted	1385871_at	-2.04
Discoidin domain receptor family, member 2	Ddr2	1389423_at	1.40
Down syndrome critical region gene 1-like 1	Dscr1l1	1398560_at	1.26
Dystrobrevin alpha (predicted)	Dtna_predicted	1380964_at	1.32
Early growth response 1	Egr1	1368321_at	-3.45
Early growth response 2	Egr2	1387306_a_at	-3.85
Early growth response 3	Egr3	1392791_at	-7.14
Enabled homolog (Drosophila)	Enah	1397449_at	-1.82
ERBB receptor feedback inhibitor 1	Errfi1	1373093_at	-2.17
Fatty acid binding protein 3	Fabp3	1376522_at	2.13
Fatty acid binding protein 5, epidermal	Fabp5	1370281_at	1.71
Fibromodulin	Fmod	1367700_at	3.09
Filamin A interacting protein 1	Filip1	1375089_at	-1.52
FK506 binding protein 3 (predicted)	Fkbp3_predicted	1392567_at	1.45
FK506 binding protein 5	Fkbp5	1388901_at	1.29
Galanin /// galanin	Gal	1387088_at	-1.43
Glutamyl aminopeptidase	Enpep	1368513_at	1.34
Glutathione peroxidase 3	Gpx3	1369926_at	1.38
Glycoprotein m6b	Gpm6b	1370389_at	1.67
GRAM domain containing 3	Gramd3	1398664_at	-1.19
Growth differentiation factor 10	Gdf10	1368459_at	1.48
Homer homolog 1 (Drosophila)	Homer1	1370454_at	-1.33
Hyaluronan synthase 2	Has2	1387548_at	-1.59

Immediate early response 2	Ier2	1372389_at	-2.13
Immediate early response 5	Ier5	1389355_at	-2.70
Immunoglobulin heavy chain (alpha polypeptide)	Igha_mapped	1374334_at	2.02
Insulin-like growth factor binding protein 6	Igfbp6	1387625_at	1.64
Mast cell protease 1	Mcpt1	1370405_at	1.47
Matrix Gla protein	Mgp	1392578_at	1.42
Microsomal glutathione S-transferase 1	Mgst1	1367612_at	1.36
Myosin, heavy polypeptide 9, non-muscle	Myh9	1387402_at	1.38
Myotubularin related protein 1 (predicted)	Mtmr1_predicted	1375885_at	-2.17
Neurotrophic tyrosine kinase, receptor, type 2	Ntrk2	1383135_at	1.42
N-myc (and STAT) interactor	Nmi	1381875_at	1.41
N-myc downstream regulated gene 1	Ndrg1	1371360_at	1.45
Nuclear receptor subfamily 4, group A, member 1	Nr4a1	1386935_at	-4.00
Nuclear receptor subfamily 4, group A, member 2	Nr4a2	1369007_at	-1.89
Nuclear receptor subfamily 4, group A, member 3	Nr4a3	1369067_at	-5.88
Osteomodulin /// osteomodulin	Omd	1387197_at	1.56
Protein kinase, cAMP dependent regulatory, type II beta	Prkar2b	1371133_a_at	1.64
Ras association (RalGDS/AF-6) domain family 4	Rassf4	1378321_at	1.53
Reelin	Reln	1373957_at	1.69
Retinol binding protein 4, plasma	Rbp4	1371762_at	1.96
Ribosomal protein L37	Rpl37	1381399_at	1.34
RT1 class Ib, locus Aw2	RT1-Aw2	1393108_at	8.45
Sclerostin domain containing 1	Sostdc1	1379281_at	-1.54
SDA1 domain containing 1	Sdad1	1390704_at	-1.28
Selenium binding protein 2	Selenbp1	1367673_at	2.25
Sema domain, immunoglobulin domain (Ig), short basic domain, secreted, (semaphorin) 3B (predicted)	Sema3b_predicted	1377336_at	1.74
Serine (or cysteine) peptidase inhibitor, clade E, member 1	Serpine1	1368519_at	-2.86
Serine (or cysteine) proteinase inhibitor, clade A (alpha-1 antiproteinase, antitrypsin), member 6	Serpina6	1373686_at	-1.75
Serum deprivation response protein	Sdpr	1382452_at	1.24
SH3-domain GRB2-like 1	Sh3gl1	1395680_at	-1.39
Signal-induced proliferation-associated 1 like 1	Sipa1l1	1395645_at	1.31
Similar to Ab2-225	RGD1306952	1372372_at	-1.45
Similar to B7-like protein GL50-B	RGD1562791_predicted	1374558_at	1.41
Similar to BTEB3 protein	RGD1565099_predicted	1383013_at	-1.19
Similar to Fam13a1 protein	RGD1309807	1393910_at	-2.13
Similar to FUN14 domain containing 2	RGD1560916_predicted	1389015_at	1.64
Similar to hypothetical protein FLJ13448	RGD1359509	1373866_at	-1.52
Similar to hypothetical protein FLJ38984	RGD1563072_predicted	1380314_at	1.30
Similar to Hypothetical protein MGC19163	RGD1309720	1384971_at	1.63
Similar to interferon-inducible GTPase /// similar to MGC108823 protein	MGC108823 /// RGD1559715_predicted	1373992_at	1.48
Similar to multimerin 1 /// similar to multimerin 1	LOC500152	1392053_at	1.64
Similar to NipSnap2 protein (Glioblastoma amplified sequence)	LOC498174	1376175_at	-1.43
Similar to OTU domain containing 1	RGD1563344_predicted	1384254_at	-3.13
Similar to Peroxisome assembly protein 10 (Peroxin-10) (Peroxisome biogenesis factor 10) (RING finger protein 69)	LOC680424	1381448_at	1.31
Similar to PWWP domain containing 2	LOC683932	1383841_at	-1.27
Similar to retinoid binding protein 7	RGD1562168_predicted	1374863_at	1.38
Similar to RIKEN cDNA 1500015O10	RGD1305645_predicted	1372646_at	2.44
Similar to RIKEN cDNA 2600010E01	RGD1309969	1376775_at	1.28
Similar to RIKEN cDNA 4832428D23 gene	RGD1561425_predicted	1380519_at	-1.28
Similar to ubiquitin-protein ligase (EC 6.3.2.19) E1 - mouse	LOC314432	1393880_at	-1.37
Similar to zinc finger protein 124	RGD1565635_predicted	1378390_at	-1.49
Solute carrier family 6 (neurotransmitter transporter, glycine), member 9	Slc6a9	1375266_at	1.41
Splicing factor, arginine/serine-rich 10 (transformer 2 homolog, Drosophila)	Sfrs10	1378122_a_at	1.39
stearoyl-Coenzyme A desaturase 2	Scd2	1367668_a_at	1.66
Tankyrase, TRF1-interacting ankyrin-related ADP-ribose polymerase 2	Tnks2_predicted	1380292_at	-1.43
Tensin like C1 domain containing phosphatase	Tenc1_predicted	1371356_at	1.35
Tetraspanin 8	Tspan8	1381125_at	1.36

Thrombospondin 1	Thbs1	1374529_at	-2.27
Transcription factor 4	Tcf4	1397286_at	-1.39
Transcription factor CP2-like 2	Tcfcp2l2	1397266_at	1.31
Transducin-like enhancer of split 1, homolog of Drosophila E(spl)	Tle1_predicted	1374425_at	-1.75
Tribbles homolog 1 (Drosophila)	Trib1	1371019_at	-1.69
Tropomyosin 3, gamma	Tpm3	1387617_at	-2.00
Ubiquitin specific protease 12 (predicted)	Usp12_predicted	1385035_at	-1.23
Ubiquitin-conjugating enzyme E2E 2	Ube2e2	1376438_at	-1.32
Zinc finger protein 278	Zfp278	1372205_at	1.23
