# Supplementary material for: Differential alterations in gene expression profiles contribute to time-dependent effects of nandrolone to prevent denervation atrophy
Source: BMC Genomics. 2010 Oct 22;11:596. doi: 10.1186/1471-2164-11-596 (PMC3091741; doi:10.1186/1471-2164-11-596)
Supplement: Additional file 2 — Genes with altered expression at 35 versus 7 days after denervation (Pool E). The file lists genes for which expression was different at 35 versus 7 days after denervation, as well as a description of each gene, its gene symbol, the name of probe set, and how much its expression changed. [file 1471-2164-11-596-S2.PDF]

Additional File 2. Genes with different expression at 35 versus 7 days after denervation (Pool E)

Description	Gene symbol	Probe set	Ratio of geom means (35 days / 7 days)
RIKEN cDNA 2810426N06 gene	2810426N06 RIK	1393175_at	1.75
alpha-2-macroglobulin	A2M	1367794_at	-2.03
ATP-binding cassette, sub-family B (MDR/TAP), member 9	ABCB9	1368484_at	-2.30
ATP-binding cassette, sub-family C (CFTR/MRP), member 1	ABCC1	1371005_at	2.97
actin binding LIM protein family, member 2	ABLIM2	1394609_at	2.60
ACN9 homolog (S. cerevisiae)	ACN9	1372847_at	-1.72
actin, alpha, cardiac muscle 1	ACTC1	1385797_at	-12.19
activin A receptor, type IIB	ACVR2B	1388179_at	2.28
ArfGAP with dual PH domains 2	ADAP2	1369725_at	2.76
adenosine deaminase, RNA-specific, B1 (RED1 homolog rat)	ADARB1	1398370_at	2.90
adenylate cyclase 9	ADCY9	1395630_at	2.96
alcohol dehydrogenase, iron containing, 1	ADHFE1	1389548_at	2.68
adiponectin receptor 2	ADIPOR2	1395146_at	-2.11
AHNAK nucleoprotein	AHNAK	1371703_at	2.19
A kinase (PRKA) anchor protein 13	AKAP13	1398369_at	1.64
A kinase (PRKA) anchor protein (yotiao) 9	AKAP9	1383151_at	1.87
akirin 1	AKIRIN1	1392908_at	-1.76
aldehyde dehydrogenase 1 family, member A1	ALDH1A1	1387022_at	1.58
angiomotin like 1	AMOTL1	1371627_at	1.65
ankyrin repeat domain 1 (cardiac muscle)	ANKRD1	1367665_at	-4.48
ankyrin repeat domain 2 (stretch responsive muscle)	ANKRD2	1384928_at	-23.81
anoctamin 1, calcium activated chloride channel	ANO1	1382387_at	4.13
amine oxidase, copper containing 3 (vascular adhesion protein 1)	AOC3	1372615_at	2.27
adaptor-related protein complex 4, sigma 1 subunit	AP4S1	1372628_at	-1.77
amyloid beta (A4) precursor protein-binding, family B, member 1 (Fe65)	APBB1	1367842_at	2.43
apolipoprotein B mRNA editing enzyme, catalytic polypeptide 1	APOBEC1	1368270_at	-1.84
aryl hydrocarbon receptor nuclear translocator-like	ARNTL	1370510_a_at	-3.07
ankyrin repeat and SOCS box-containing 5	ASB5	1391924_at	-2.95
aspartate beta-hydroxylase	ASPH	1381046_at	-2.13
asp (abnormal spindle) homolog, microcephaly associated (Drosophila)	ASPM	1393581_at	-1.69
asporin	ASPN	1381504_at	-4.74
activating transcription factor 5	ATF5	1372601_at	2.53
Bardet-Biedl syndrome 2	BBS2	1368509_at	2.02
bobby sox homolog (Drosophila)	BBX	1383829_at	1.82
3-hydroxybutyrate dehydrogenase, type 2	BDH2	1372613_at	2.27
basic helix-loop-helix family, member e41	BHLHB3	1368511_at	3.17
BMP binding endothelial regulator	BMPER	1391345_at	3.59
BTG family, member 2	BTG2	1386995_at	-5.35
chromosome 11 open reading frame 17	C11ORF17	1383175_a_at	-2.08
chromosome 1 open reading frame 216	C1ORF216	1380314_at	3.04
complement component 1, q subcomponent, B chain	C1QB	1370215_at	-1.52

C1q and tumor necrosis factor related protein 2	C1QTNF2	1382185_at	2.16
chromosome 7 open reading frame 36	C7ORF36	1392504_at	-2.24
chromosome 9 open reading frame 89	C9ORF89	1374333_at	-1.95
calcium channel, voltage-dependent, beta 1 subunit	CACNB1	1378073_at	1.46
calcyclin binding protein	CACYBP	1392979_at	-2.53
calmodulin-like 3	CALML3	1384836_at	33.99
calsequestrin 2 (cardiac muscle)	CASQ2	1387401_at	-6.81
cyclin A2	CCNA2	1379582_a_at	-1.72
cyclin B2	CCNB2	1389566_at	-2.17
CD68 molecule	CD68	1375010_at	-1.85
cell division cycle 2-like 6 (CDK8-like)	CDC2L6	1374066_at	1.93
CDV3 homolog (mouse)	CDV3	1383028_at	-4.049
CCAAT/enhancer binding protein (C/EBP), delta	CEBPD	1387343_at	2.05
carboxylesterase 1 (monocyte/macrophage serine esterase 1)	CES1 (includes EG:1066)	1370363_at	3.01
coiled-coil-helix-coiled-coil-helix domain containing 3	CHCHD3	1389291_at	-1.81
checkpoint with forkhead and ring finger domains	CHFR	1393293_at	-1.86
chitinase 3-like 1 (cartilage glycoprotein-39)	CHI3L1	1392171_at	-2.70
cytidine monophosphate (UMP-CMP) kinase 2, mitochondrial	CMPK2	1383424_at	2.42
2',3'-cyclic nucleotide 3' phosphodiesterase	CNP	1387897_at	3.50
contactin 2 (axonal)	CNTN2	1387424_at	5.27
collagen, type XIV, alpha 1	COL14A1	1376105_at	-2.06
collagen, type V, alpha 2	COL5A2	1370895_at	-2.31
ceruloplasmin (ferroxidase)	CP	1368418_a_at	2.33
cysteine sulfinic acid decarboxylase	CSAD	1398286_at	1.91
cytotoxic T lymphocyte-associated protein 2 alpha	CTLA2A	1389659_at	-1.56
cullin 3	CUL3	1393343_at	1.72
D site of albumin promoter (albumin D-box) binding protein	DBP	1387874_at	3.07
discoidin domain receptor tyrosine kinase 2	DDR2	1389423_at	2.33
de-etiolated homolog 1 (Arabidopsis)	DET1	1376950_at	-2.28
dehydrogenase E1 and transketolase domain containing 1	DHTKD1	1390172_at	2.55
Dmx-like 1	DMXL1	1382340_at	2.26
DnaJ (Hsp40) homolog, subfamily C, member 6	DNAJC6	1377125_at	-2.56
dual specificity phosphatase 11 (RNA/RNP complex 1-interacting)	DUSP11	1376576_at	-1.54
dynein, light chain, Tctex-type 1	DYNLT1	1386882_at	-1.58
early growth response 2 (Krox-20 homolog, Drosophila)	EGR2	1387306_a_at	-6.85
EH-domain containing 3	EHD3	1391442_at	1.96
eukaryotic translation initiation factor 2C, 2	EIF2C2	1368867_at	-2.20
eukaryotic translation initiation factor 4E family member 2	EIF4E2	1373876_at	-2.58
eukaryotic translation initiation factor 5	EIF5	1388206_a_at	-2.07
enabled homolog (Drosophila)	ENAH	1396450_at	-2.25
ecto-NOX disulfide-thiol exchanger 2	ENOX2	1384198_at	1.35
predicted gene, ENSMUSG00000062038	ENSMUSG00000062038	1387773_at	-1.94
erythrocyte membrane protein band 4.1 (elliptocytosis 1, RH-linked)	EPB41	1392607_at	3.91
EPH receptor B3	EPHB3	1385788_at	1.94
v-erb-b2 erythroblastic leukemia viral oncogene homolog 2, neuro/glioblastoma derived oncogene homolog (avian)	ERBB2	1387813_at	4.08
eukaryotic translation termination factor 1	ETF1	1389038_at	-1.71
v-ets erythroblastosis virus E26 oncogene homolog 2 (avian)	ETS2	1372564_at	1.58

coagulation factor III (thromboplastin, tissue factor)	F3	1369182_at	2.24
fatty acid desaturase 1	FADS1	1367857_at	3.24
family with sequence similarity 124A	FAM124A	1378339_at	1.62
family with sequence similarity 126, member B	FAM126B	1397259_at	3.34
family with sequence similarity 129, member B	FAM129B	1388482_at	2.43
family with sequence similarity 45, member B	FAM45B	1375829_at	-2.85
family with sequence similarity 96, member B	FAM96B	1372001_at	-2.11
fibroblast activation protein, alpha	FAP	1369422_at	-1.53
fatty acyl CoA reductase 1	FAR1	1382629_at	-2.62
fibrillin 1	FBN1	1387351_at	-2.32
Fc fragment of IgG, low affinity IIa, receptor (CD32)	FCGR2A	1398246_s_at	-3.95
four and a half LIM domains 1	FHL1	1394750_at	-4.55
FK506 binding protein 5	FKBP5	1388901_at	2.29
fibronectin 1	FN1	1370234_at	-2.43
follistatin	FST	1387843_at	3.03
FXYD domain containing ion transport regulator 6	FXYD6	1370248_at	8.70
galanin prepropeptide	GAL	1387088_at	-2.21
growth associated protein 43	GAP43	1367930_at	-2.44
guanine nucleotide binding protein (G protein), beta 5	GNB5	1382105_at	1.82
guanine nucleotide binding protein (G protein), gamma 12	GNG12	1377739_at	-2.35
golgi membrane protein 1	GOLM1	1390050_at	2.18
glycoprotein (transmembrane) nmb	GPNMB	1368187_at	-4.05
G protein-coupled receptor 177	GPR177	1383129_at	2.28
glutathione peroxidase 3 (plasma)	GPX3	1369926_at	1.87
growth factor receptor-bound protein 10	GRB10	1397211_at	2.90
glutathione S-transferase mu 1	GSTM1	1370952_at	3.03
glutathione S-transferase mu 2 (muscle)	GSTM2	1387023_at	2.74
glycophorin C (Gerbich blood group)	GYPC	1372273_at	1.93
hyaluronan synthase 2	HAS2	1387548_at	-2.19
histone deacetylase 4	HDAC4	1376761_at	1.33
hemochromatosis type 2 (juvenile)	HFE2	1391429_at	2.34
major histocompatibility complex, class I, C	HLA-C	1371078_at	2.95
homer homolog 2 (Drosophila)	HOMER2	1368872_a_at	-2.46
HRAS-like suppressor	HRASLS	1393084_at	2.28
hydroxysteroid (11-beta) dehydrogenase 1	HSD11B1	1386953_at	-1.93
heat shock 27kDa protein family, member 7 (cardiovascular)	HSPB7	1392435_at	-2.93
inhibitor of DNA binding 4, dominant negative helix-loop-helix protein	ID4	1385923_at	1.35
immediate early response 2	IER2	1372389_at	-2.04
immediate early response 5	IER5	1389355_at	-2.21
immunoglobulin superfamily, member 6	IGSF6	1387687_at	-1.71
interleukin 11 receptor, alpha	IL11RA	1370331_at	2.24
InaD-like (Drosophila)	INADL (includes EG:10207)	1385043_at	1.93
IQ motif and ubiquitin domain containing	IQUB	1376124_at	2.25
integrin, alpha 9	ITGA9	1380236_at	4.28
potassium inwardly-rectifying channel, subfamily J, member 11	KCNJ11	1387698_at	1.78
kinesin-associated protein 3	KIFAP3	1389556_at	1.80
Kruppel-like factor 15	KLF15	1368249_at	2.05
Kruppel-like factor 5 (intestinal)	KLF5	1385961_at	2.25

Kruppel-like factor 7 (ubiquitous)	KLF7	1377618_at	2.00
karyopherin alpha 1 (importin alpha 5)	KPNA1	1382251_at	-2.72
keratin 18	KRT18	1388155_at	3.38
laminin, beta 2 (laminin S)	LAMB2	1367880_at	2.58
LIM and cysteine-rich domains 1	LMCD1	1376632_at	-4.27
LIM domain 7	LMO7	1381798_at	3.72
lysyl oxidase	LOX	1368172_a_at	-4.51
lysyl oxidase-like 1	LOXL1	1388902_at	-2.08
lysyl oxidase-like 4	LOXL4	1376435_at	1.99
latent transforming growth factor beta binding protein 2	LTBP2	1368448_at	-1.73
lymphocyte antigen 6 complex, locus E	LY6E	1388347_at	1.77
lymphatic vessel endothelial hyaluronan receptor 1	LYVE1	1382192_at	-1.88
v-maf musculoaponeurotic fibrosarcoma oncogene homolog F (avian)	MAFF	1380229_at	-2.33
mannan-binding lectin serine peptidase 1 (C4/C2 activating component of Ra-reactive factor)	MASP1	1389543_at	1.51
matrin 3	MATR3	1382522_at	2.30
malic enzyme 3, NADP(+)-dependent, mitochondrial	ME3	1389572_at	2.60
Meis homeobox 1	MEIS1	1384308_at	1.93
mediator of cell motility 1	MEMO1 (includes EG:51072)	1389312_at	-2.24
mex-3 homolog B (C. elegans)	MEX3B	1380682_at	-1.92
MAX gene associated	MGA (includes EG:23269)	1391600_at	2.40
mannosyl (alpha-1,6-)-glycoprotein beta-1,2-N-acetylglucosaminyltransferase	MGAT2	1386982_at	-1.54
hypothetical protein LOC84792	MGC12966	1378399_at	-1.78
microsomal glutathione S-transferase 1	MGST1	1367612_at	2.34
MICAL-like 2	MICALL2	1379059_at	1.92
myeloid/lymphoid or mixed-lineage leukemia (trithorax homolog, Drosophila)	MLL	1381069_at	2.38
myeloid/lymphoid or mixed-lineage leukemia 5 (trithorax homolog, Drosophila)	MLL5	1384125_at	1.75
myeloid/lymphoid or mixed-lineage leukemia (trithorax homolog, Drosophila); translocated to, 11	MLLT11	1371692_at	-2.95
myeloid/lymphoid or mixed-lineage leukemia (trithorax homolog, Drosophila); translocated to, 3	MLLT3	1368279_at	-3.46
membrane protein, palmitoylated 6 (MAGUK p55 subfamily member 6)	MPP6	1380062_at	-2.84
mannose receptor, C type 1	MRC1	1392648_at	-2.36
mitochondrial ribosomal protein L24	MRPL24	1371888_at	-1.60
musashi homolog 2 (Drosophila)	MSI2	1392572_at	-2.33
macrophage scavenger receptor 1	MSR1	1382601_at	-2.65
metallothionein 1E	MT1E	1388271_at	-4.33
metallothionein 1F	MT1F	1388267_a_at	-2.05
5,10-methenyltetrahydrofolate synthetase (5-formyltetrahydrofolate cyclo-ligase)	MTHFS	1381768_at	-1.87
musculoskeletal, embryonic nuclear protein 1	MUSTN1	1373032_at	-2.27
MAX interactor 1	MXI1	1372093_at	1.90
myosin IE	MYO1E	1370933_at	1.61
myocilin, trabecular meshwork inducible glucocorticoid response	MYOC	1387313_at	2.47
myogenic differentiation 1	MYOD1	1398655_at	2.10
NEDD4 binding protein 1	N4BP1	1391222_at	-1.86
N-acetyltransferase 13 (GCN5-related)	NAT13	1398382_at	1.76
nuclear receptor coactivator 1	NCOA1	1390010_at	1.97

neuroepithelial cell transforming 1	NET1	1372000_at	2.17
nuclear factor of activated T-cells 5, tonicity-responsive	NFAT5	1398421_at	1.63
nuclear factor of kappa light polypeptide gene enhancer in B-cells inhibitor, alpha	NFKBIA	1389538_at	4.58
N-myc (and STAT) interactor	NMI	1381875_at	2.01
NODAL modulator 1	NOMO1	1388852_at	1.72
NIPA-like domain containing 2	NPAL2	1390474_at	2.36
neuropeptide Y receptor Y1	NPY1R	1390828_at	2.22
nuclear receptor subfamily 4, group A, member 1	NR4A1	1386935_at	-2.53
neurotrophic tyrosine kinase, receptor, type 2	NTRK2	1397246_at	3.33
NudC domain containing 1	NUDCD1	1393753_at	-2.17
nudix (nucleoside diphosphate linked moiety X)-type motif 4	NUDT4	1398847_at	-3.62
osteomodulin	OMD	1387197_at	2.14
purinergic receptor P2X, ligand-gated ion channel, 6	P2RX6	1368740_at	3.52
palmdelphin	PALMD	1376924_a_at	-2.17
pannexin 1	PANX1	1382934_at	-1.95
POZ (BTB) and AT hook containing zinc finger 1	PATZ1	1372205_at	2.34
polycomb group ring finger 6	PCGF6	1389760_at	-2.08
pericentriolar material 1	PCM1	1371064_at	1.73
PDZ and LIM domain 2 (mystique)	PDLIM2	1375367_at	3.30
PDS5, regulator of cohesion maintenance, homolog B (S. cerevisiae)	PDS5B	1392891_at	1.91
period homolog 3 (Drosophila)	PER3	1378745_at	4.00
phosphoinositide-3-kinase adaptor protein 1	PIK3AP1	1376795_at	2.22
phosphoinositide-3-kinase, class 2, beta polypeptide	PIK3C2B	1381576_at	2.50
PTEN induced putative kinase 1	PINK1	1372199_at	1.81
plakophilin 4	PKP4	1372744_at	2.50
plasminogen activator, urokinase receptor	PLAUR	1387269_s_at	-2.20
phospholipase B1	PLB1	1370447_at	-2.02
phospholipase C, delta 1	PLCD1	1367916_at	2.13
phospholamban	PLN	1370157_at	1.91
periplakin	PPL	1391187_at	4.80
protein phosphatase 1J (PP2C domain containing)	PPM1J	1374310_at	-1.91
protein phosphatase 1, regulatory (inhibitor) subunit 3C	PPP1R3C	1395236_at	-1.84
protein regulator of cytokinesis 1	PRC1	1392899_at	-1.93
prolyl endopeptidase-like	PREPL	1374004_at	-2.30
protein kinase, AMP-activated, beta 2 non-catalytic subunit	PRKAB2	1376724_at	1.89
protein kinase, cAMP-dependent, regulatory, type II, beta	PRKAR2B	1371133_a_at	2.08
proline rich 12	PRR12	1392593_a_at	2.17
paired related homeobox 1	PRRX1	1384840_at	2.40
proteasome (prosome, macropain) activator subunit 4	PSME4	1387345_at	-1.95
prostaglandin-endoperoxide synthase 2 (prostaglandin G/H synthase and cyclooxygenase)	PTGS2	1368527_at	-3.24
protein tyrosine phosphatase-like (proline instead of catalytic arginine), member A	PTPLA	1383696_at	-2.95
protein tyrosine phosphatase, receptor type, C	PTPRC	1390798_at	-2.21
protein tyrosine phosphatase, receptor type, D	PTPRD	1395148_at	6.96
RAB15, member RAS onocogene family	RAB15	1397839_at	-1.53
RAB21, member RAS oncogene family	RAB21	1383089_at	-1.82
RAB, member of RAS oncogene family-like 4	RABL4	1388927_at	1.84
Rac GTPase activating protein 1	RACGAP1	1373658_at	-2.09
Ras association (RalGDS/AF-6) and pleckstrin homology domains 1	RAPH1	1374525_at	1.99

RNA binding motif protein 39	RBM39	1381967_at	1.78
RNA binding motif protein 5	RBM5	1398595_at	2.79
regulator of calcineurin 1	RCAN1	1388686_at	-5.05
regulator of calcineurin 2	RCAN2	1389066_at	4.15
RCSD domain containing 1	RCSD1	1373903_at	2.42
RAS-like, estrogen-regulated, growth inhibitor	RERG	1390812_a_at	3.19
Rho-related BTB domain containing 3	RHOBTB3	1389003_at	-1.58
ras homolog gene family, member C	RHOC	1371659_at	-1.95
Rho GTPase-activating protein	RICS	1377061_at	1.78
RIO kinase 3 (yeast)	RIOK3	1395699_at	-2.32
replication protein A1, 70kDa	RPA1	1372181_at	1.74
ribosomal protein L3	RPL3	1396049_x_at	-1.84
Ras-related GTP binding D	RRAGD	1373427_at	-2.75
related RAS viral (r-ras) oncogene homolog 2	RRAS2	1382058_at	-2.56
ribonucleotide reductase M2 polypeptide	RRM2	1389408_at	-2.82
Rtf1, Paf1/RNA polymerase II complex component, homolog (S. cerevisiae)	RTF1	1377667_at	1.86
RUN and FYVE domain containing 3	RUFY3	1378194_a_at	-3.08
sterile alpha motif domain containing 8	SAMD8	1393932_at	-2.43
sodium channel, voltage-gated, type III, beta	SCN3B	1383435_at	-2.16
syndecan 2	SDC2	1370167_at	-1.94
serologically defined colon cancer antigen 8	SDCCAG8	1372874_at	1.96
SEC14-like 1 (S. cerevisiae)	SEC14L1	1397334_at	-1.75
sema domain, immunoglobulin domain (Ig), short basic domain, secreted, (semaphorin) 3B	SEMA3B	1377336_at	1.86
secretion regulating guanine nucleotide exchange factor	SERGEF	1390755_at	1.77
serpin peptidase inhibitor, clade E (nexin, plasminogen activator inhibitor type 1), member 1	SERPINE1	1392264_s_at	-1.79
splicing factor 3a, subunit 1, 120kDa	SF3A1	1389328_at	1.84
single-minded homolog 2 (Drosophila)	SIM2	1385722_at	2.17
solute carrier family 12 (sodium/potassium/chloride transporters), member 2	SLC12A2	1367853_at	2.09
solute carrier family 16, member 1 (monocarboxylic acid transporter 1)	SLC16A1	1386981_at	-1.47
solute carrier family 25 (mitochondrial carrier; Graves disease autoantigen), member 16	SLC25A16	1384368_at	-2.17
solute carrier family 35 (UDP-N-acetylglucosamine (UDP-GlcNAc) transporter), member A3	SLC35A3	1395589_at	-2.09
SAFB-like, transcription modulator	SLTM	1381175_at	2.80
SWI/SNF related, matrix associated, actin dependent regulator of chromatin, subfamily a, member 2	SMARCA2	1385074_at	1.95
SWI/SNF related, matrix associated, actin dependent regulator of chromatin, subfamily d, member 2	SMARCD2	1370159_at	1.87
small nuclear ribonucleoprotein polypeptide F	SNRPF	1379542_at	-1.63
sorbin and SH3 domain containing 1	SORBS1	1375349_at	2.33
SRY (sex determining region Y)-box 4	SOX4	1384000_at	-2.13
spastic paraplegia 7 (pure and complicated autosomal recessive)	SPG7	1374839_at	1.64
spindlin 1	SPIN1	1376195_at	-1.89
spire homolog 1 (Drosophila)	SPIRE1	1396081_at	-2.34
spermidine synthase	SRM	1367834_at	-1.92
single stranded DNA binding protein 3	SSBP3	1368517_at	1.84
sarcospan (Kras oncogene-associated gene)	SSPN	1380563_at	2.42
ST3 beta-galactoside alpha-2,3-sialyltransferase 2	ST3GAL2	1381528_at	-1.98
signal transducing adaptor family member 2	STAP2	1389420_at	3.47
stathmin 1/oncoprotein 18	STMN1	1386857_at	-1.68

STE20-related kinase adaptor beta	STRADB	1375896_at	-1.87
transcription elongation factor A (SII), 3	TCEA3	1388611_at	1.72
transcription elongation factor B (SIII), polypeptide 3 (110kDa, elongin A)	TCEB3	1389778_a_at	-1.80
thyrotrophic embryonic factor	TEF	1390819_at	2.25
tensin like C1 domain containing phosphatase (tensin 2)	TENC1	1371356_at	2.34
transferrin receptor (p90, CD71)	TFRC	1371113_a_at	-11.24
TGFB-induced factor homeobox 1	TGIF1	1373421_at	-2.75
translocase of inner mitochondrial membrane 8 homolog A (yeast)	TIMM8A	1368400_at	-2.37
TIMP metallopeptidase inhibitor 3	TIMP3	1389836_a_at	2.50
tumor necrosis factor receptor superfamily, member 11b	TNFRSF11B	1369407_at	-2.53
tumor necrosis factor receptor superfamily, member 12A	TNFRSF12A	1371785_at	-4.02
tumor necrosis factor receptor superfamily, member 21	TNFRSF21	1391573_at	2.26
TNFAIP3 interacting protein 1	TNIP1	1388492_at	1.83
troponin T type 3 (skeletal, fast)	TNNT3	1397499_at	-2.28
tenascin XA	TNXA	1388145_at	1.94
transducer of ERBB2, 2	TOB2	1375677_at	2.93
topoisomerase (DNA) II alpha 170kDa	TOP2A	1388650_at	-1.68
tropomyosin 3	TPM3	1371184_x_at	-3.05
tubulin polymerization-promoting protein family member 3	TPPP3	1398365_at	-1.80
tribbles homolog 1 (Drosophila)	TRIB1	1371019_at	-2.45
TSC22 domain family, member 3	TSC22D3	1367771_at	2.10
TSC22 domain family, member 4	TSC22D4	1371550_at	3.28
tetratricopeptide repeat domain 27	TTC27	1374227_at	2.57
tubulin, alpha 4a	TUBA4A	1371542_at	-3.95
thioredoxin-like 1	TXNL1	1368936_at	-1.57
ubiquitin-conjugating enzyme E2C	UBE2C	1388484_at	-1.70
ubiquitin-conjugating enzyme E2Q family member 2	UBE2Q2	1393072_at	-1.99
ubiquitin family domain containing 1	UBFD1	1382396_at	-1.79
ubiquitin-like 4A	UBL4A	1392906_at	-2.48
UBX domain protein 2A	UBXN2A	1382067_at	-3.61
uridine-cytidine kinase 2	UCK2	1383945_at	-2.56
ubiquitin specific peptidase 12	USP12	1385035_at	-2.18
ubiquitin specific peptidase 54	USP54	1372356_at	1.63
vasohibin 2	VASH2	1398426_at	2.77
versican	VCAN	1388142_at	-3.86
vestigial like 2 (Drosophila)	VGLL2	1384033_at	-1.54
very low density lipoprotein receptor	VLDLR	1387455_a_at	-3.00
vacuolar protein sorting 33 homolog A (S. cerevisiae)	VPS33A	1389626_at	2.00
vacuolar protein sorting 36 homolog (S. cerevisiae)	VPS36	1389191_at	-2.07
zinc finger and BTB domain containing 20	ZBTB20	1394849_at	1.92
zinc finger, DHHC-type containing 3	ZDHHC3	1390039_at	-2.19
zinc finger, MIZ-type containing 1	ZMIZ1	1389927_at	2.20
zinc finger protein 367	ZNF367	1379967_at	-2.67
zinc finger protein 446	ZNF446	1391702_at	1.76
