# Supplementary material for: Differential alterations in gene expression profiles contribute to time-dependent effects of nandrolone to prevent denervation atrophy
Source: BMC Genomics. 2010 Oct 22;11:596. doi: 10.1186/1471-2164-11-596 (PMC3091741; doi:10.1186/1471-2164-11-596)
Supplement: Additional file 3 — Genes altered by nandrolone at 35 days (Pool B). The file lists genes for which expression was altered by nandrolone at 35 days as well as a description of each gene, its gene symbol, the name of probe set, and how much its expression changed. [file 1471-2164-11-596-S3.PDF]

Additional File 3. Genes altered by nandrolone at 35 days (Pool B)

Description	Gene symbol	Probe set	Ratio of geom means
AE binding protein 1 (predicted)	Aebp1_predicted	1395695_at	3.44
Apolipoprotein D	Apod	1398258_at	14.73
AT hook, DNA binding motif, containing 1 (predicted)	Ahdc1_predicted	1380858_at	-1.92
ATPase, Ca++ transporting, plasma membrane 1	Atp2b1	1397224_at	-1.45
B-cell translocation gene 3	Btg3	1368072_at	1.32
Brain-specific angiogenesis inhibitor 2 (predicted)	Bai2_predicted	1378871_at	-1.82
Calcium/calmodulin-dependent protein kinase II, alpha	Camk2a	1388187_at	-3.23
calcineurin B, type I	Ppp3r1	1369152_at	2.11
Camello-like 1	Cml1	1368208_at	-1.49
Carboxypeptidase A3	Cpa3	1371151_at	1.77
Casein kinase 1, alpha 1	Csnk1a1	1394689_at	-2.44
Cd36 antigen	Cd36	1367689_a_at	2.23
CDC23 (cell division cycle 23, yeast, homolog)	Cdc23	1395640_at	1.69
CDNA clone IMAGE:7321089		1389986_at	-2.13
ChaC, cation transport regulator-like 1 (E. coli) (predicted)	Chac1_predicted	1389573_at	-1.69
Chemokine (C-C motif) ligand 7	Ccl7	1379935_at	1.70
Chemokine (C-C motif) receptor 1	Ccr1	1370083_at	1.55
Clusterin	Clu	1367784_a_at	2.52
Complement component 1, s subcomponent	C1s	1387893_at	2.28
Complement component factor H	Cfh	1387029_at	1.73
CTD (carboxy-terminal domain, RNA polymerase II, polypeptide A) small phosphatase 1	Ctdsp1	1373320_at	-1.54
CTD (carboxy-terminal domain, RNA polymerase II, polypeptide A) small phosphatase-like (predicted)	Ctdspl_predicted	1379238_at	1.57
Cytochrome b-245, beta polypeptide	Cybb	1379344_at	2.21
developmentally regulated GTP binding protein 1	Drg1	1391602_at	3.97
Dicer1, Dcr-1 homolog (Drosophila) /// Calmin (predicted)	Dicer1	1378089_at	-2.00
Discoidin domain receptor family, member 2	Ddr2	1368673_at	-1.56
DNA-damage-inducible transcript 4-like	Ddit4l	1368013_at	-1.72
Down syndrome critical region gene 1-like 1	Dscr1l1	1389066_at	-1.69
Early growth response 1	Egr1	1392653_at	2.28
Early growth response 3	Egr3	1392791_at	2.29
Ect2 oncogene (predicted)	Ect2_predicted	1383747_at	1.62
Ectonucleoside triphosphate diphosphohydrolase 4 (predicted)	Entpd4_predicted	1375728_at	-1.61
EGL nine homolog 3 (C. elegans)	Egln3	1368174_at	-1.64
Elastin	Eln	1388111_at	1.46
Endothelial differentiation sphingolipid G-protein-coupled receptor 1	Edg1	1396233_at	1.61
Epithelial membrane protein 1	Emp1	1369736_at	3.35
Eukaryotic elongation factor-2 kinase	Eef2k	1389999_at	-2.44
Folate receptor 2 (fetal) (predicted)	Folr2_predicted	1390348_at	1.53
Follistatin-like 1	Fstl1	1368821_at	2.43
Forkhead box M1	Foxm1	1392058_at	-1.56
Forkhead box protein O1A	FOXO1	1396965_at	-2.22
G0/G1 switch gene 2	G0s2	1388395_at	2.07
GNAS complex locus	Gnas	1387906_a_at	-2.38
Golgi associated, gamma adaptin ear containing, ARF binding protein 2	Gga2	1395394_at	1.52
Grainyhead-like 1 (Drosophila) (predicted)	Grhl1_predicted	1378128_at	-2.70
Growth arrest and DNA-damage-inducible 45 gamma	Gadd45g	1388792_at	-2.13
Guanine nucleotide binding protein, alpha 12	Gna12	1369278_at	1.94
Heat shock transcription factor 4 (predicted)	Hsf4_predicted	1377288_at	-1.72

Hook homolog 3 (Drosophila)	Hook3	1390467_at	-1.39
Human immunodeficiency virus type I enhancer binding protein 1	Hivep1	1391560_at	1.72
Hydroxysteroid 11-beta dehydrogenase 1 /// tetraspanin 8	Hsd11b1 /// Tspan8	1368052_at	-1.85
Hypothetical LOC303211	RGD1311260	1375430_at	-1.39
Hypothetical LOC310764 (predicted)	RGD1306526_predicted	1377305_at	-1.92
Hypothetical protein LOC619558	LOC619558	1381940_at	-1.89
Integrin beta 1 (fibronectin receptor beta)	Itgb1	1387346_at	1.75
Integrin beta 2	Itgb2	1383131_at	1.69
Ischemia related factor vof-16	Vof16	1377778_at	-3.03
Junction adhesion molecule 2	Jam2	1392309_at	2.45
Kallikrein 7	Klk7	1387820_at	-1.82
Kinesin 2	Kns2	1370886_a_at	-1.82
Lysyl oxidase	Lox	1368171_at	2.44
Myelin basic protein	Mbp	1368810_a_at	12.06
Myosin binding protein H	Mybph	1368966_at	-1.82
Myotrophin	Mtpn	1387786_at	1.92
Nephroblastoma overexpressed gene	Nov	1368883_at	1.91
Neural precursor cell expressed, developmentally down-regulated gene 1 (predicted)	Nedd1_predicted	1379457_at	-2.17
Nuclear protein 1	Nupr1	1367847_at	2.17
Osteoglycin (predicted)	Ogn_predicted	1383263_at	2.63
Oxysterol binding protein-like 2	Osbpl2	1378814_at	-2.38
Peptidylprolyl isomerase (cyclophilin)-like 4 (predicted)	Ppil4_predicted	1396171_at	1.45
Period homolog 1 (Drosophila)	Per1	1374855_at	-1.54
Phosphatidylserine synthase 2 (predicted)	Ptdss2_predicted	1396267_at	1.98
Plastin 3 (T-isoform)	Pls3	1371139_at	2.11
Procollagen, type XII, alpha 1	Col12a1	1398321_a_at	1.76
Procollagen-proline, 2-oxoglutarate 4-dioxygenase (proline 4-hydroxylase), alpha polypeptide III	P4ha3	1393129_at	2.65
Prosaposin	Psap	1383409_at	-2.04
Protein kinase inhibitor, alpha	Pkia	1368982_at	2.47
Protein kinase, AMP-activated, a1 caltalic unit	Prkaa1	1369104_at	1.93
Protein kinase, AMP-activated, gamma 3 non-catatlytic subunit (predicted)	Prkag3_predicted	1394711_at	-1.64
Protein phosphatase 1, catalytic subunit, beta isoform	Ppp1cb	1392730_at	-1.92
PWWP domain containing 2 (predicted)	Pwwp2_predicted	1389800_at	-1.30
Receptor accessory protein 6	Reep6	1384639_at	1.45
Regulator of G-protein signaling 2	Rgs2	1368144_at	1.96
Ring finger protein (C3H2C3 type) 6 (predicted)	Rnf6_predicted	1380651_at	-1.82
secreted frizzled-related protein 2	Sfrp2	1390119_at	2.36
Secreted frizzled-related protein 4	Sfrp4	1368394_at	2.48
Sequestosome 1	Sqstm1	1375374_at	-1.92
Serine (or cysteine) peptidase inhibitor, clade A (alpha-1 antiproteinase, antitrypsin), member 12	Serpina12	1379065_at	-2.27
Similar to AP2 associated kinase 1 (predicted)	RGD1563580_predicted	1376459_at	-1.89
Similar to asporin precursor	LOC306805	1380726_at	3.92
Similar to Breast carcinoma amplified sequence 3 homolog (K20D4) (predicted)	RGD1560788_predicted	1389603_at	-1.92
Similar to ENSANGP00000020885 (predicted)	RGD1563825_predicted	1379411_at	-1.92
Similar to fructosamine-3-kinase (predicted)	Fnsk_predicted	1383654_a_at	-1.92
Similar to hypothetical protein 3010020C06	RGD1310893_predicted	1390906_at	-1.92
similar to hypothetical protein FLJ23033	RGD1566117_predicted	1394422_at	-1.89
similar to KIAA0605 gene product	RGD1305459_predicted	1393060_at	-1.85
Similar to KIAA1217	RGD1563437_predicted	1397152_at	-2.22
similar to Mob4B protein	MGC124888	1392961_at	1.72
similar to muscleblind-like 1 isoform d	LOC686892	1394931_at	1.98
similar to Protein CXorf17 homolog	RGD1564253_predicted	1394459_at	-2.13
similar to RIKEN cDNA 2810428I15	RGD1566239_predicted	1372746_at	-1.69
Similar to RIKEN cDNA 5033405K12	RGD1311593_predicted	1380752_at	-1.72
similar to smoothelin, like	LOC679629	1377695_at	-1.54
similar to solute carrier family 25, member 36	LOC501039	1392789_at	1.63
Similar to T-cell activation protein phosphatase 2C (predicted)	RGD1310383_predicted	1375498_at	-1.79
Similar to transcription factor RAM2	LOC619566	1398216_at	-1.72

Similar to transmembrane protein TM9SF	RGD1564625_predicted	1380053_at	1.55
Similar to Tribbles homolog 2	RGD1564451_predicted	1388821_at	1.45
Solute carrier family 30 (zinc transporter), member 2	Slc30a2	1398264_at	-1.96
Solute carrier family 43, member 2	Slc43a2_predicted	1384756_at	-2.08
Solute carrier family 9 (sodium/hydrogen exchanger), isoform 3 regulator 1	Slc9a3r1 /// RGD1308513	1375672_at	-1.89
Sortilin 1	Sort1	1391230_at	-1.82
Sprouty protein with EVH-1 domain 1, related sequence	Spred1	1377743_at	2.93
stearoyl-Coenzyme A desaturase 1	Scd1	1370355_at	2.51
T-cell immunomodulatory protein	Cda08	1382448_at	-1.64
Testis expressed gene 2	Tex2	1380885_at	-2.13
Thrombospondin 4	Thbs4	1388138_at	1.58
Transducer of ERBB2,2	Tob2	1375677_at	-1.43
Transforming, acidic coiled-coil containing protein 2	Tacc2	1378055_at	-2.38
Transmembrane protein 14A (predicted)	Tmem14a_predicted	1391684_at	2.01
Transmembrane protein 2 (predicted)	Tmem2_predicted	1394160_at	-2.08
Transmembrane protein 49	Tmem49	1370807_at	1.31
TruB pseudouridine (psi) synthase homolog 1 (E. coli)	Trub1	1382151_at	-1.67
TSC22-related-inducible leucine zipper protein 2	TSC22	1367771_at	-1.19
